# Transcriptome and proteome dynamics in chemostat culture reveal how *Campylobacter jejuni* modulates metabolism, stress responses and virulence factors upon changes in oxygen availability

**DOI:** 10.1111/1462-2920.13930

**Published:** 2017-10-02

**Authors:** Edward J. Guccione, John J. Kendall, Andrew Hitchcock, Nitanshu Garg, Michael A. White, Francis Mulholland, Robert K. Poole, David J. Kelly

**Affiliations:** ^1^ Department of Molecular Biology and Biotechnology The University of Sheffield, Firth Court Western Bank Sheffield S10 2TN UK; ^2^ Institute of Food Research, Norwich Research Park Colney Lane Norwich NR4 7UA UK

## Abstract

*Campylobacter jejuni*, the most frequent cause of food‐borne bacterial gastroenteritis worldwide, is a microaerophile that has to survive high environmental oxygen tensions, adapt to oxygen limitation in the intestine and resist host oxidative attack. Here, oxygen‐dependent changes in *C*. *jejuni* physiology were studied at constant growth rate using carbon (serine)‐limited continuous chemostat cultures. We show that a perceived aerobiosis scale can be calibrated by the acetate excretion flux, which becomes zero when metabolism is fully aerobic (100% aerobiosis). Transcriptome changes in a downshift experiment from 150% to 40% aerobiosis revealed many novel oxygen‐regulated genes and highlighted re‐modelling of the electron transport chains. A label‐free proteomic analysis showed that at 40% aerobiosis, many proteins involved in host colonisation (e.g., PorA, CadF, FlpA, CjkT) became more abundant. PorA abundance increased steeply below 100% aerobiosis. In contrast, several citric‐acid cycle enzymes, the peptide transporter CstA, PEB1 aspartate/glutamate transporter, LutABC lactate dehydrogenase and PutA proline dehydrogenase became more abundant with increasing aerobiosis. We also observed a co‐ordinated response of oxidative stress protection enzymes and Fe‐S cluster biogenesis proteins above 100% aerobiosis. Our approaches reveal key virulence factors that respond to restricted oxygen availability and specific transporters and catabolic pathways activated with increasing aerobiosis.

## Introduction

*Campylobacter jejuni* is a human pathogen of enormous public health and environmental significance. It is the leading cause of acute bacterial gastroenteritis worldwide and is acquired predominantly by ingesting contaminated food, milk or water (O'Brien, 2017). Ecologically, the bacterium is widely distributed in bird species, with poultry (particularly chicken) serving as the primary source of human infection (Sheppard *et al*., 2009). Preventative biosecurity measures aimed at reducing environmental contamination and exposure of chickens to the pathogen have so far been largely ineffective. Although often self‐limiting, human infection can be associated with severe sequelae, including Guillain‐Barré and other syndromes. There are currently no widely‐used vaccines or *Campylobacter*‐resistant chickens available and new insights into *C*. *jejuni* ecophysiology, environmental survival and host interactions are needed if control measures are to be put in place that reduce chicken colonisation and thus food‐chain contamination.

Although *C*. *jejuni* has the ability to survive in a variety of environmental niches outside of avian or mammalian hosts, the factors contributing to its adaptability are poorly understood. A key distinction in the physiology of *C*. *jejuni* compared to many other enteric bacteria is that it is a classical microaerophilic bacterium, unable to grow at normal atmospheric oxygen tensions on agar plates or in shaken cultures, but adapted to host niches that contain low oxygen concentrations (Krieg and Hoffman, 1986; Kendall *et al*., 2014). Microaerophily is therefore one of the major defining features of the biology of this food‐borne pathogen. During its life cycle, *C*. *jejuni* will be exposed to highly variable oxygen concentrations, and it presents an interesting paradox; although oxygen sensitive, it must be able to survive high environmental oxygen tensions, resist the oxidative stresses encountered *in vivo*, and adapt to the severe oxygen limitation of the gastro‐intestinal tract. In the intestine, there is a steep oxygen gradient across the mucosal layer. Although campylobacters are invasive, a large population grows in the viscous mucosal matrix, where the oxygen concentration is likely to be very low. Understanding the mechanisms of adaptation to oxygen limitation is thus key to elucidating how *C*. *jejuni* causes disease. *Campylobacter jejuni* is a respiratory bacterium with a surprisingly complex branched electron transport chain for a host‐adapted, small genome pathogen (Kelly, 2008). Oxygen‐dependent respiration is catalysed by two terminal oxidases of differing oxygen affinity (Jackson *et al*., 2007). The cytochrome *c* oxidase (CcoNOQP) is of the *cbb_3_* type, often found in microaerophiles, and has a very high oxygen affinity (*K*_d_ ∼ 40 nM) that may allow growth at lower oxygen levels than the alternative *bd*‐like (CioAB or CydAB) quinol oxidase (*K*_d_ ∼ 8 µM; Jackson *et al*., 2007). Indeed, 1‐day old chick colonisation experiments have shown that while a *cydA* mutant colonised as well as the wild‐type, a *ccoN* mutant was completely unable to colonise the chick caecum (Weingarten *et al*., 2008). Most strains of *C*. *jejuni* also possess a wide range of terminal reductases allowing energy conservation with alternative electron acceptors which may be present in the host and other environments, such as nitrate, nitrite, trimethylamine‐N‐oxide, dimethylsulphoxide, fumarate and tetrathionate (Sellars *et al*., 2002; Pittman *et al*., 2007; Weingarten *et al*., 2009; Guccione *et al*., 2010; Liu *et al*., 2013). There is some evidence for a role for nitrate and nitrite respiration *in vivo* (Weingarten *et al*., 2008). However, little is known about how oxygen regulates the expression of these various electron transport chains, apart from the role of the RacRS system, which curtails fumarate reduction in the presence of the energetically preferred electron acceptor nitrate under low oxygen conditions (van der Stel *et al*., 2015).

How campylobacters respond to higher oxygen conditions, is of interest in relation to the nature of microaerophily, their survival in the environment and host colonisation. What is clear is that *C*. *jejuni* is not deficient in oxidative stress defence enzymes (reviewed by Flint *et al*., 2016) with most strains possessing catalase, superoxide dismutase, three cytoplasmic peroxiredoxins (AhpC, Tpx and Bcp), two cytoplasmic methionine sulphoxide reductases, two periplasmic cytochrome *c* peroxidases, a desulforuberythrin and several hemerythrins (Parkhill *et al*., 2000; Flint *et al*., 2016). In addition, comprehensive mutagenesis studies have revealed other enzymes and proteins that also seem to play a role in the destruction of reactive oxygen species (ROS) or in other mechanisms of oxidative stress defence (Flint *et al*., 2014). *C*. *jejuni* does lack the well characterized regulators of the oxidative stress response found in other bacteria, such as OxyR and SoxRS (Parkhill *et al*., 2000), but is now known to possess its own unique set of regulatory proteins that control the expression of several of the above enzymes in response to ROS. These include the essential OmpR‐type response regulator CosR (Hwang *et al*., 2011), PerR (Butcher *et al*., 2015) and two MarR‐type regulators designated RrpA and RrpB (Gundogdu *et al*., 2015) in addition to the iron‐responsive regulator Fur which also regulates oxidative stress related genes (Butcher *et al*., 2015).

Although *C*. *jejuni* seems well equipped to deal with oxidative stress using similar enzymatic mechanisms to those found in many other bacteria, most strains remain oxygen‐sensitive. One explanation is that, compared to conventional aerobes, *C*. *jejuni* is unique in utilizing oxidant‐labile enzymes in central metabolic pathways, which are critical for growth. In particular, it employs flavodoxin dependent 2‐oxoacid oxidoreductases, rather than oxygen‐stable NAD‐linked dehydrogenases, to transfer substrate derived electrons to the respiratory chain (Kendall *et al*., 2014; Weerakoon and Olson, 2008). These enzymes, pyruvate:acceptor oxidoreductase (POR) and 2‐oxoglutarate:acceptor oxidoreductase (OOR) contain Fe‐S clusters vulnerable to oxidative damage; exposure of *C*. *jejuni* cells to prolonged aeration has been shown to cause their inactivation *in vivo* and this has been proposed as a major contributor to the microaerophilic phenotype of these bacteria (Kendall *et al*., 2014). Individual strains of *C*. *jejuni* do show wide variation in their oxygen tolerance, however, and some remarkably aerotolerant strains have recently been described (Oh *et al*., 2015; Rodrigues *et al*., 2015). This seems to be related to an increased resistance to ROS in such strains, mediated by AhpC for example (Oh *et al*., 2015).

The way in which oxygen regulates gene expression and protein synthesis at a global level in *C*. *jejuni* is poorly understood. Previous studies have used either liquid batch or plate cultures exposed to atmospheres with various oxygen contents (Gaynor *et al*., 2004; Sulaeman *et al*., 2012; van der Stel *et al*., 2017), sometimes generated within gas jars. Two major drawbacks can be identified with this approach. First, in batch cultures the population growth rate will change with variation in oxygen availability and it is extremely difficult to separate effects of oxygen *per se* on gene/protein expression from those resulting from growth rate or growth phase changes. Second, the actual oxygen availability experienced by the cells will crucially depend on the oxygen transfer rate and the cells respiration rate, and may be very different from the atmosphere above the culture. These factors are compounded by arbitrary choices by the experimenter of whether the cultures are shaken or not, the shaking speed, the medium composition and the length of time of growth before sampling. These factors make it virtually impossible to compare results from different laboratories.

In this article, we use a combination of transcriptomic, proteomic and biochemical techniques to assess how global gene and protein expression changes with oxygen availability in cells grown in continuous chemostat culture at a series of defined steady‐states at different degrees of aerobiosis. This is complemented by studies of the response over time to the transition from a high to a low oxygen regime, which mimics the journey of campylobacters from the environment into the intestine of the host. The power of this approach lies in the use of nutrient‐limited chemostat culture with continuous gas sparging, where at steady‐state the cell population is growing exponentially at a fixed, specific growth rate and where the oxygen availability to each cell is (on average) the same, but can be varied independently, with all other parameters kept constant. Thus, the effects of oxygen can be much more precisely defined than is ever possible in batch culture. Accordingly, we have identified genes that were previously not known to be oxygen‐regulated, including many related to colonization and growth in the host. The data provide a foundation for the identification of the sensory and transcriptional circuits that underlie these responses.

## Results

### Steady‐state physiological parameters for the growth of *C. jejuni* in continuous culture at a range of oxygen supply rates

**Figure 1:**
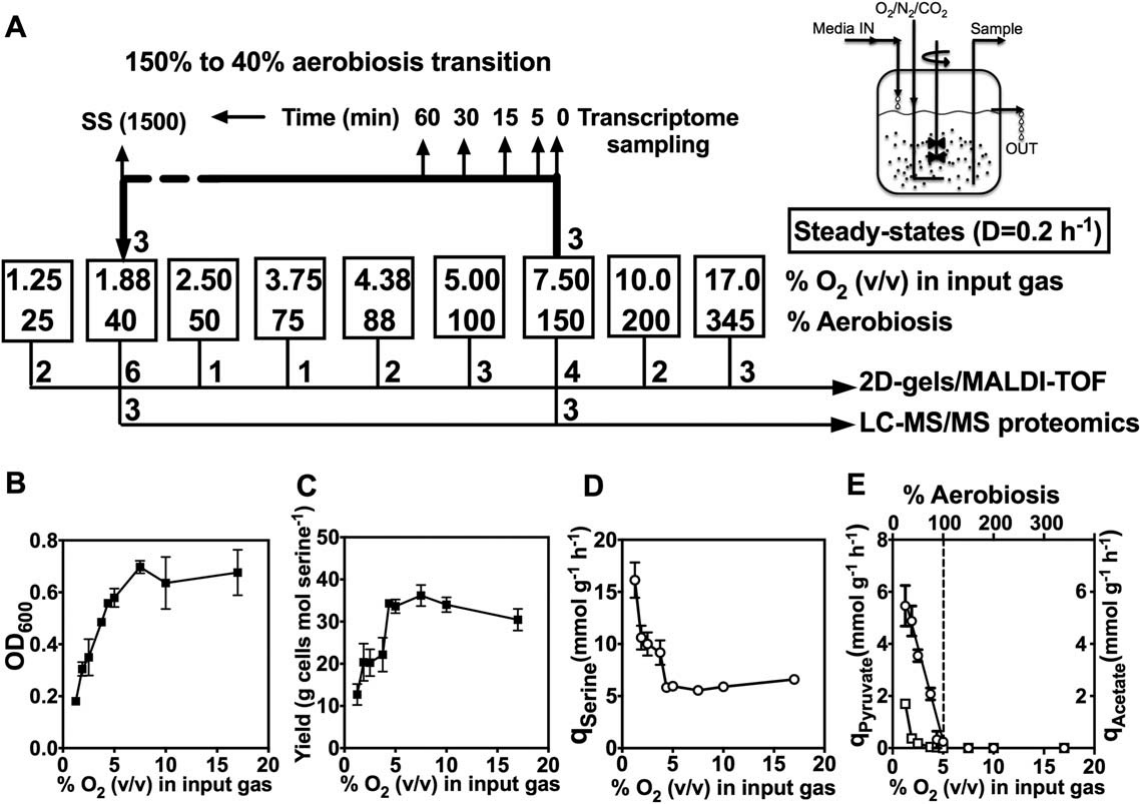
Experimental design (A) and physiological parameters of *C*. *jejuni* growth in continuous culture (B–E). In (A), nine different conditions of oxygen availability were established in the chemostat by altering the composition of the input gas mixture. The boxed numbers show the % (v/v) of oxygen in the input gas and the corresponding degree of aerobiosis as calculated from the acetate excretion rate of the cells at steady‐state (panel E). A variable number of independent steady‐states derived from separate inocula were established at each of the nine oxygen input conditions, which were sampled for proteomics analysis by 2D‐gels and MALDI‐TOF MS of selected tryptically digested spots as described in Materials and Methods. The number of separate steady‐states used for this analysis is shown below the boxes. Three independent steady‐states at 150% and 40% aerobiosis were also subjected to LC‐MS/MS analysis after 1D‐gel separation for a more extensive proteome coverage. After steady‐states had been reached at 150% aerobiosis, the gas input was reduced to 1.88% (v/v) oxygen (40% aerobiosis). Samples from three such experiments were taken at different times during the transition as shown, for transcriptomic analysis by microarray analysis. Panel (B) shows the mean optical density at each steady‐state, panel (C) shows the mean growth yield on l‐serine at each steady‐state (determined from the cell dry weight and the amount of serine consumed), panel (D) shows the serine consumption rate (*q*_Serine_) and panel (E) shows the specific pyruvate (open squares) and acetate (open circles) excretion rates (*q*_pyruvate_ and *q*_Acetate_). The linear relationship between *q*_Acetate_ and the oxygen input was used to define the aerobiosis scale, where *q*_Acetate_ = 0 = 100% aerobiosis. In (B–E), the errors bars show standard deviation of the mean.

For a rigorous investigation of the influence of oxygen on cell physiology, the ability to fix the growth rate of the cells while varying oxygen availability in the absence of variation of other environmental parameters is a key requirement. This can be achieved in a chemostat, where the specific growth rate (μ) depends on the rate of supply of a single growth‐limiting nutrient, defined by the dilution rate (*D*), such that at steady‐state *D* = μ (Pirt, 1975). In this study, we chose to use carbon limitation, where under conditions of sufficient aeration, all of the carbon source supplied to the culture will be converted to biomass and carbon dioxide (Pirt, 1975). *Campylobacter jejuni* NCTC 11168 was grown in a bespoke defined minimal medium in which the exogenously supplied N‐source was ammonium sulphate and l‐serine was the major carbon (and additional nitrogen) source. Preliminary experiments showed that low concentrations of several other amino acids had to be included in this medium to allow growth in the chemostat without the culture washing out. However, it should be noted that *C*. *jejuni* NCTC 11168 can only catabolise serine, aspartate, glutamate and proline (Guccione *et al*., 2008); l‐proline was included at < 1 mM and aspartate and glutamate were absent, so growth is essentially entirely dependent on l‐serine. We established steady‐states in the chemostat at nine different oxygen inputs in the gas‐flow over the range 1.25% v/v to 17% v/v oxygen by sparging through the medium at a constant gas flow and stirring rate (Fig. 1A). The dilution rate was set at 0.20 h^−1^ (3.5 h doubling time; *D* = μ = ln2/*t*_d_ in steady‐state, where *t*_d_ is the doubling time), in each case. A minimum of two and a maximum of seven independent steady‐states from fresh inocula were sampled at each different oxygen input condition (Fig. 1A). Above 17% v/v oxygen in the gas‐flow, we could not establish a stable steady‐state and the culture washed out of the chemostat. Between 4% v/v and 17% v/v oxygen in the gas‐flow the optical density and yield of cells on serine remained relatively constant (Fig. 1B,C), while below 4% v/v oxygen these parameters decreased and the specific rate of serine consumption (*q*_serine_) increased (Fig. 1D) as acetate was excreted (Fig. 1E). ^1^H‐NMR analysis of culture supernatants obtained at each steady‐state showed complete utilisation of the growth limiting substrate l‐serine at all oxygen inputs from 17% v/v to 2.5% v/v inclusive, while at 1.88% v/v and 1.25% v/v, residual l‐serine concentrations of ∼0.3 mM and ∼1.6 mM were recorded respectively. Thus, at 1.25% v/v oxygen in the gas‐flow, the cells are likely to be oxygen‐limited rather than carbon‐limited; in order to determine if growth was still carbon‐limited at 1.88% v/v oxygen in the gas‐flow, we established a steady‐state, turned off the media pump and added a bolus of 10 mM l‐serine to the culture vessel. As expected for a carbon‐limited culture, the optical density increased steadily after the addition (from 0.32 to 0.42 over 200 min). We also performed a washout experiment (Pirt, 1975) at this oxygen input condition, to determine the maximum specific growth rate (*μ*_max_). This was determined to be 0.22 h^−1^, 10% above the set dilution rate. No culture instability was noted, suggesting that *D* = 0.2 h^−1^ is below the critical dilution rate (*D*_c_). The NMR analyses showed that acetate was by far the major exometabolite excreted by the cells when the oxygen supply was reduced below 5% v/v in the gas‐flow; surprisingly, we also found that some pyruvate was excreted at very low oxygen inputs (Fig. 1E). Pyruvate is the immediate deamination product resulting from the action of serine dehydratase. No lactate or other overflow metabolites were detected at any steady‐state.

### A perceived aerobiosis scale based on the acetate excretion flux

Oxygen‐electrode measurements of the residual dissolved oxygen tensions (rDOT) in chemostat cultures of bacteria show distinctly non‐linear relationships with the oxygen‐supply rates, due to the homeostatic respiratory activity response of the biomass (Pirt, 1975; Alexeeva *et al*., 2002). Thus, rDOT is not a practically useful measure of the oxygen status of the cells. An alternative is to define the minimal oxygen input that is needed for fully aerobic catabolism i.e. complete oxidation of the carbon source to cells plus CO_2_; this is equivalent to ‘100% aerobiosis’ (Alexeeva *et al*., 2002). For *E*. *coli* in glucose‐limited chemostat cultures, it was shown that 100% aerobiosis can be quantified as the point at which the acetate excretion flux (*q*_Acetate_) is zero; from 100% to 0% aerobiosis, *q*_Acetate_ was found to increase linearly and can thus be used to calibrate an aerobiosis scale (Alexeeva *et al*., 2002). We tested this approach with *C*. *jejuni* NCTC 11168. We found that, as with *E*. *coli*, a linear relationship could be shown between the oxygen supply rate and *q*_Acetate_ in our serine‐limited cultures, while *q*_Pyruvate_ appeared non‐linear and much less sensitive (Fig. 1E). This allowed us to define 100% aerobiosis as 5 ± 0.4% v/v oxygen in the gas‐flow in our chemostat set‐up (Fig. 1E). We were unable to define 0% aerobiosis experimentally as we could not establish steady‐states below 1.25% v/v oxygen in the gas‐flow without washout (*C*. *jejuni* cannot ferment l‐serine and no exogenous electron acceptors were present in our experiments to allow oxygen‐independent respiration); extrapolation to the *y*‐axis in Fig. 1E gave a *q*_Acetate_ value of ∼7.5 mmol g cells^−1^ h^−1^ at 0% aerobiosis. Thus, in our chemostat system, increasing degrees of microaerobic growth occur at steady‐states of less than about 5% v/v oxygen in the gas‐flow, while above that concentration, the cells metabolism is fully aerobic. We used the aerobiosis scale in this study to compare changes in gene expression and protein synthesis with oxygen availability.

### Transient and steady‐state transcriptomic and proteomic analyses: Experimental design and overview

In order to compare global gene expression under fully aerobic versus microaerobic conditions, we conducted a microarray analysis at two steady‐state conditions of oxygen availability in the chemostat; 150% aerobiosis (7.5% oxygen v/v in the gas‐flow in our system) and 40% aerobiosis (1.88% v/v oxygen in the gas‐flow in our system), with three independently established steady‐states at each condition (see Fig. 1A). In addition, after the 150% aerobiosis steady‐states were established and sampled, the input gas mixture was changed to 40% aerobiosis and samples taken at timed intervals after the down‐shift (Fig. 1A) to capture transient changes accompanying a change in oxygen availability. Supporting Information Fig. S1 shows the physiological data for the transition experiments. Both the optical density and dry weight decreased between 0 and 300 min after the shift but the cell viability remained relatively constant. Within 5 min after the shift, acetate could be detected in the culture supernatant and *q*_Acetate_ increased steadily during the transition (Supporting Information Fig. S1). Samples taken at 5, 15, 30 and 60 mins, along with samples from the 150% and 40% aerobiosis steady‐states, were processed for microarray analysis (See Fig. 1A and *Experimental procedures*). The full microarray dataset for the 150% aerobiosis steady‐states compared to the transition samples and 40% aerobiosis steady‐states is given in Supporting Information Table S1 and the raw data are available at ArrayExpress with the accession number E‐MTAB‐5743. Five selected genes that were either up‐regulated or down‐regulated during the transition according to the microarray data were also shown to have the same pattern of regulation by RT‐PCR (Supporting Information Table S1). Additional validation for a range of genes is provided by the proteomic analyses described below. Supporting Information Fig. S2 gives an overall picture of the gene categories that showed greater than twofold changes in expression. It is clear that in terms of numbers, genes involved in cell envelope functions, energy metabolism, protein synthesis and transport and binding‐proteins represented the major classes that were either up‐ or down‐regulated. Moreover, the highest percentage of genes changing in expression was in the energy metabolism category, followed by the cell envelope category. Transient down‐regulation of many of the ribosomal protein genes was observed during the transition (Supporting Information Table S1), suggesting a decrease in protein synthesis capacity before the cells adapted to the new steady‐state. Other specific gene expression changes are discussed in detail below.

To complement the gene expression data, a label‐free LC‐MS/MS proteomic analysis was also carried out on cells from the steady‐states at 150% and 40% aerobiosis (Fig. 1A, Supporting Information Table S2). A quantitative analysis based on the exponentially modified protein abundance index (emPAI) using spectral counting (Ishihama *et al*., 2005) with high stringency levels at 99.9% for the Peptide Threshold and 99.9% plus at least two peptides for the Protein Threshold, gave 857 proteins identified with a high level of confidence. Using a statistical cut‐off of *p* = 0.02, we identified 223 proteins that were more highly abundant at 40% aerobiosis and 129 that were more highly abundant at 150% aerobiosis, while 505 proteins were not significantly changed between the two conditions (Supporting Information Table S2).

Analysis of protein abundance at just two steady‐state oxygen availabilities does not allow a complete picture of the patterns of synthesis of different proteins to be obtained. Therefore, a series of additional steady‐states over the range of 25–345% aerobiosis were established (Fig. 1A), cell samples were harvested, cell‐free extracts prepared and subjected to 2D‐gel electrophoresis as described in *Experimental procedures*. The full raw dataset for this second type of proteomic analysis is given in Supporting Information Table S3. This method cannot give such deep proteome coverage as the label‐free analysis shown in Supporting Information Table S2 and a significant number of spots were composed of more than one protein (and therefore could not be used in this analysis). Nevertheless, the number of individual protein spots identified was broad enough to allow responses of key functional groups of enzymes and other proteins to oxygen availability to be determined.

### Re‐modelling of the electron transport chains below and above 100% aerobiosis

**Figure 2:**
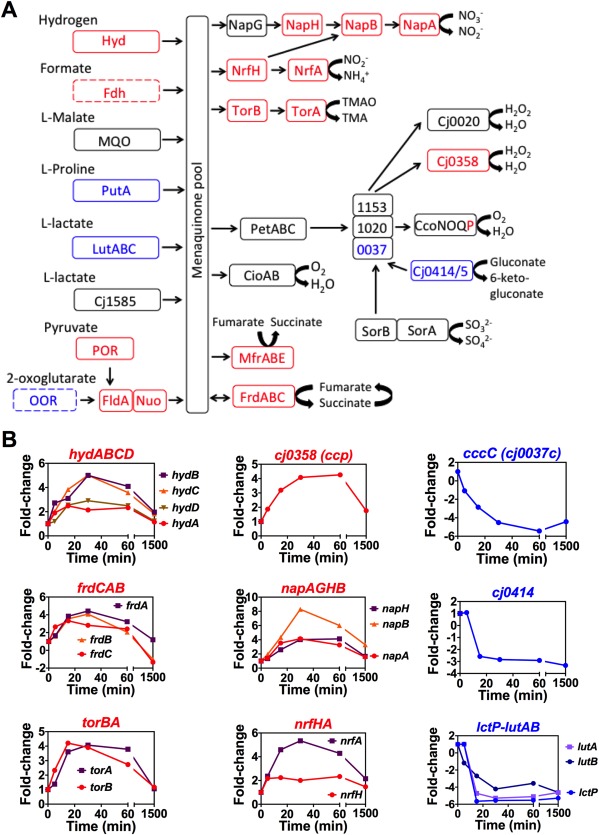
Changes in expression of genes encoding electron transport proteins during the 150% to 40% aerobiosis transition. A. Schematic overview of the major pathways of electron transport in *C*. *jejuni* NCTC 11168. Proteins encoded by genes up‐regulated twofold or more during the transition from 150% to 40% aerobiosis are shown in red, those proteins encoded by genes down‐regulated twofold or more are shown in blue, while those not significantly changed in expression are shown in black. Dotted lines indicate enzymes with smaller fold‐changes but where additional evidence for the regulatory pattern shown has been obtained (see text). B. Individual expression dynamics of selected genes, derived from the dataset in Supporting Information Table S1. Each panel shows the change in expression normalised to a onefold change at 150% aerobiosis (time 0). Most genes show a transient change in expression before falling back to an expression level characteristic of the new steady‐state.

Figure 2A provides a current view of the electron transport chain structure of *C*. *jejuni* NCTC 11168, based on our previous work (Sellars *et al*., 2002; Pittman *et al*., 2007; Hitchcock *et al*., 2010; Liu and Kelly, 2015). The gene regulation patterns during the 150–40% aerobiosis transition are indicated in red (upregulated) or blue (downregulated) to give a picture of the most important changes. Overall, a clear pattern was observed of the up‐regulation of genes encoding components of alternative respiratory pathways to oxygen and of genes encoding the hydrogenase enzyme for the utilisation of hydrogen (*hydABCD*), a very low midpoint redox potential electron donor (*E*_m7_ < −400 mV) that would be important *in vivo* under low oxygen conditions. The label‐free proteomics also confirmed a greater abundance of the hydrogenase large and small subunits (HydA and HydB) at 40% aerobiosis (Supporting Information Table S2). Although we did not observe a significant change in formate dehydrogenase subunit gene (*fdhABC*) expression or FdhA protein synthesis (Supporting Information Tables S1 and S2), the expression of *cj1514c* encoding the FdhA specific chaperone FdhM (Hitchcock *et al*., 2010) was increased up to fourfold during the transition. Fdh is a seleno‐enzyme; we showed that the apparent lack of regulation of formate dehydrogenase structural genes was due to the fact that our standard defined medium did not contain added selenium (Supporting Information Fig. S3A). When additional steady‐states were established in the presence of sodium selenate, a greater than twofold increase in formate dependent oxygen‐linked respiration was observed at 40% compared to 150% aerobiosis (Supporting Information Fig. S3A).

In contrast, some other genes encoding dehydrogenase enzymes acting on electron donors of less negative redox potential were down‐regulated in the transition to low oxygen. Most strikingly, the proline utilisation A (*putA*) gene was very strongly downregulated (Figs 2 and 6A) and is discussed separately in detail below. The genes encoding the lactate permease LctP and one of the two respiratory lactate dehydrogenases in *C*. *jejuni* (LutABC) encoded by *cj0783‐cj0785* (Thomas *et al*., 2011) were also strongly decreased in expression in the high to low oxygen transition. In each case, the proteomics analysis showed an increased abundance of the cognate proteins at 150% versus 40% aerobiosis (Supporting Information Table S2), consistent with the gene expression data. l‐lactate respiration rates were not significantly different at high and low aerobiosis (Supporting Information Fig. S3B), likely reflecting the contribution of the unregulated alternative l‐lactate dehydrogenase Cj1585 to this activity (Thomas *et al*., 2011). The flavodoxin gene *fldA* and the *nuo* genes encoding the flaxodoxin:quinone oxidoreductase (Weerakoon and Olson, 2008) were slightly up‐regulated in the transition (Supporting Information Table S1).

*Campylobacter jejuni* can also use certain high redox potential electron donors that feed electrons into the respiratory chain at the level of cytochromes *c*, presumably only allowing the use of oxygen as the electron acceptor (Fig. 2A). In strain NCTC 11168, these include sulphite and gluconate; their respective dehydrogenase genes *sorAB* (*cj0005c/cj0004c*) and *cj0414/415* were unchanged and downregulated respectively in the shift experiment (Fig. 2B). Sulphite dependent respiration rates were not significantly different at low or high aerobiosis (Supporting Information Fig. S3E). The 2D‐gel analysis (Supporting Information Table S3) showed that Cj0414 was increased 15‐fold at high aerobiosis (345% compared to 75% aerobiosis) and Cj0415 was increased a maximum of 10‐fold (345% compared to 25% aerobiosis), consistent with an oxygen‐respiration linked physiological role (Fig. 2B). At least three soluble periplasmic cytochromes *c* have been shown to have a role in the transfer of electrons between the cytochrome *bc_1_* complex and the *cbb_3_*‐type cytochrome *c* oxidase (Liu and Kelly, 2015); these are CccA (Cj1153), CccB (Cj1020) and CccC (Cj0037). Interestingly, the cognate genes for these cytochromes were differentially regulated in response to oxygen, suggesting distinct functions. *cj1153* (*cccA*) was slightly up‐regulated during the shift from 150% to 40% aerobiosis (by ∼2.5‐fold) but CccA protein levels were not significantly different in the label‐free proteomics analysis (Supporting Information Table S2) or on a haem‐stained gel (Supporting Information Fig. S4). The expression of *cccB* was also not significantly altered during the transition. In contrast, *cj0037c* (*cccC*) was strongly down‐regulated (greater than fivefold) and the CccC protein was correspondingly 23.5‐fold more abundant at 150% versus 40% aerobiosis (Supporting Information Table S2), suggesting a specific oxygen‐linked role, perhaps as the preferred electron donor to the cytochrome *c* oxidase (Liu and Kelly, 2015).

The *cj0081/82* genes encoding the cytochrome *bd*‐like menaquinol oxidase (CydAB/CioAB) were not significantly changed in expression from the microarray data, but the label‐free proteomics analysis showed that the CydA protein was 7.4‐fold more abundant at 40% aerobiosis compared to 150% and CydB was only detected in the 40% aerobiosis samples (Supporting Information Table S2). The *ccoP* gene of the *cbb_3_*‐type cytochrome *c* oxidase (CcoNOQP) was the only gene of this complex that showed an expression change during the transition to low oxygen (Supporting Information Table S1), but CcoP and CcoO were only slightly (1.6‐ and 1.9‐fold respectively) more abundant at 40% aerobiosis (Supporting Information Table S2). Measurements of cytochrome *c* oxidase activity confirmed there was no significant difference in cells grown at the high and low aerobiosis conditions (Supporting Information Fig. S3F).

The individual dynamics of the gene expression changes for the *frd, nap, nrf* and *tor* operons, (encoding reductase enzymes for fumarate, nitrate, nitrite and TMAO/DMSO respectively) during the shift from aerobic to microaerobic metabolism are shown in Fig. 2B. All of these genes were upregulated in response to lower oxygen availability, but it is apparent that a comparison of the two steady‐states gives an incomplete impression of the extent of the regulatory patterns and that much larger transient changes accompany the shift from 150% to 40% aerobiosis. These data were very well correlated with the label free proteomic analysis (Supporting Information Table S2), which showed that at 40% aerobiosis the menaquinol:fumarate reductase/succinate dehydrogenase subunits (FrdABC), nitrate reductase (NapAB), nitrite reductase (NrfAH) and the TMAO/DMSO reductase (TorAB; Cj0264/Cj0265) proteins were all significantly more abundant than at 150% aerobiosis. Apart from FrdABC, these enzymes all have integral cytochrome *c* electron transferring or catalytic subunits. On a haem‐stained gel, the increased abundance of the pentahaem NrfA and the tetrahaem NrfH is very apparent at low versus high aerobiosis (Supporting Information Fig. S4) supporting the proteomic data. Interestingly, three enzymes of haem biosynthesis, HemA, HemL and HemN, and the cytochrome *c* synthase CcsBA (Cj1013; Liu and Kelly, 2015) were 2.1‐fold, 1.8‐fold, fourfold and 1.8‐fold higher at 40% aerobiosis respectively (Supporting Information Table S2), in keeping with an extra demand for cytochrome *c* biogenesis under these conditions.

### *mfrABE* gene expression, protein abundance and enzyme activity is strongly increased below 100% aerobiosis in a RacR dependent manner

**Figure 3:**
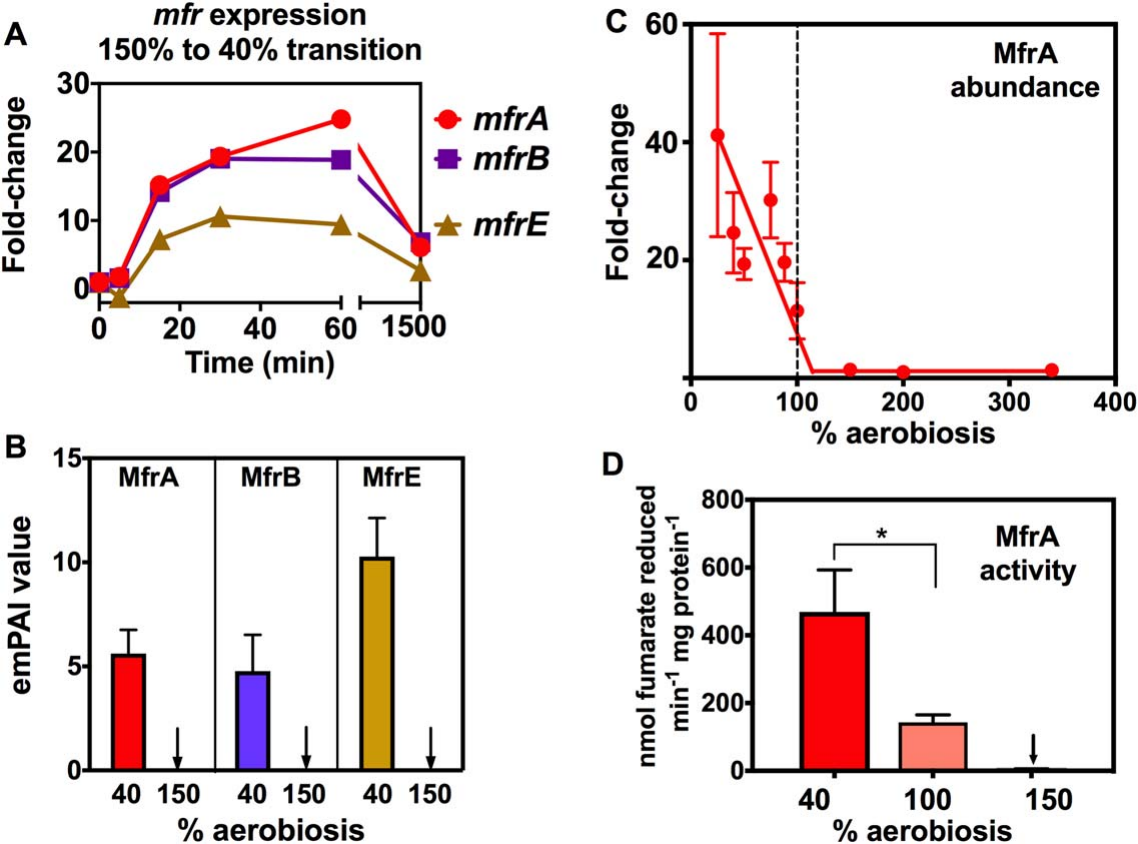
Methylmenaquinol:fumarate reductase (Mfr) gene expression, protein subunit abundance and enzyme activity changes markedly in response to oxygen availability. A. Gene expression changes during the 150% to 40% aerobiosis transition (normalised to onefold at 150% aerobiosis), showing large transient fold‐changes for the genes encoding the active‐site flavoprotein subunit (*mfrA*), the electron transferring iron‐sulphur protein (*mfrB*) and the membrane anchor containing the methylmenaquinol oxidation site (*mfrE*). B. Relative abundance of MfrA, MfrB and MfrE in steady‐states at 40% and 150% aerobiosis, measured by LC‐MS/MS. C. Correlation of MfrA abundance at each steady‐state (expressed as the fold‐change in the normalised spot volume from 2D‐gel analysis; raw data in Supporting Information Table S3) with enzyme activity (D) measured in periplasmic extracts as the rate of fumarate dependent oxidation of reduced methyl viologen (* *p* < 0.05 by Students *t*‐test). In each panel, error bars represent standard deviation of the mean.

The largest fold‐change for any gene in the microarray data was for *mfrA*, encoding the active site flavoprotein subunit of the unusual periplasmic facing methylmenaquinol:fumarate reductase MfrABE (previously mis‐annotated as succinate dehydrogenase SdhABC; Guccione *et al*., 2010). This enzyme is restricted to epsilonproteobacteria and is thought to be important for rapid adaptation to fumarate respiring conditions; it also has activity with the fumarate analogues mesaconate and crotonate, which are produced by intestinal anaerobes (Guccione *et al*., 2010). Sixty minutes after the shift from 150% to 40% aerobiosis, *mfrA* was upregulated a maximum of ∼25‐fold before falling back to approximately sixfold in the 40% aerobiosis steady‐state (Fig. 3A). The co‐transcribed genes for the other subunits of the enzyme, *mfrB* and *mfrE*, followed a similar pattern. In the label‐free proteomic analyses, all three subunits of the enzyme were detected at 40% aerobiosis but not at 150% aerobiosis (Fig. 3B) and the 2D‐gel analysis over the whole range of aerobiosis used in this work clearly showed the dramatic effect of oxygen on the abundance of MfrA, which steeply increased below 100% aerobiosis (Fig. 3C). These changes were also highly correlated with the enzyme activity of MfrA, measured as fumarate dependent reduced methyl viologen oxidation in periplasmic extracts (Fig. 3D).

In response to low oxygen in the presence of alternative electron acceptors of favourable redox potential, such as nitrate (Em_7_ nitrate/nitrite = +420 mV) or TMAO (Em_7_ TMAO/TMA = +130 mV) the two‐component RacRS system is activated to repress expression of genes involved in less energetically favourable fumarate respiration (Em_7_ fumarate/succinate = +30 mV), including *mfr, aspA* and *dcuA* (van der Stel *et al*., 2015). However, our experiments were conducted in media that did not contain any added alternative electron acceptors to oxygen, so the transcriptomic data suggest *mfrABE* and other members of the RacR regulon are oxygen‐ or redox‐regulated via RacRS. Probabilistic modelling using the program TFINFER (Asif *et al*., 2010; see *Experimental Procedures*), which combines connectivity data about the structure of the known RacR regulatory network (van der Stel *et al*., 2015) with the time‐course microarray data, implied an approximately twofold decrease in RacR activity up to 60 min post‐shift, followed by an increase towards the new 40% aerobiosis steady‐state (Supporting Information Fig. S5A). The modelled regulatory strengths (Supporting Information Fig. S5B) show the biggest influence of RacR is on *mfrA* and *mfrB* expression, with smaller effects on *aspA* and *dcuA*, *cj0448c* (*tlp6*) and *cj0358* (*ccpA2*) expression. The data suggest that upon a reduction of oxygen availability, even in the absence of alternative electron acceptors, RacR is involved, directly or indirectly, in increased transcription of these genes.

### Increased abundance of specific colonisation and virulence proteins occurs below 100% aerobiosis

**Figure 4:**
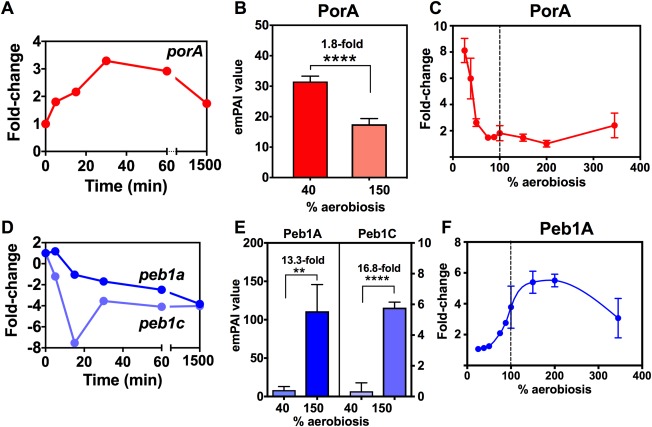
Oxygen‐dependent changes in gene expression and protein abundance of the MOMP/PorA (A–C) and Peb1 ABC‐transporter (D–F). In (A) the time‐dependent increase in *porA* expression during the shift from 150% to 40% aerobiosis steady‐states is shown. (B) The difference in PorA abundance at the two steady‐states measured by label‐free LC‐MS/MS. (C) The fold‐change in PorA abundance at all steady‐states as measured on 2D‐gels (raw data in Supporting Information Table S3). The same plots are shown in (D–F) for the *peb1a* and *peb1c* genes (**D**) and proteins (**E** and **F**). ** *p* < 0.01, **** *p* < 0.0001 by Students *t*‐test. Error bars represent standard deviation of the mean.

Inspection of the label‐free proteomic data at 150% and 40% aerobiosis revealed that many proteins involved in host colonisation and/or interaction, were more abundant at lower oxygen availability (Supporting Information Table S2). A clear example of a key virulence factor that we found to be oxygen‐regulated was the major outer membrane protein (MOMP) encoded by the *porA* gene (Fig. 4A–C). During the 150–40% aerobiosis transition, *porA* expression transiently increased to a maximum of ∼3.3‐fold by 30 min (Fig. 4A). The label‐free analysis showed a significantly higher abundance of PorA at 40% compared to 150% aerobiosis (Fig. 4B) and the 2D‐gel analysis (Fig. 4C) showed a dramatic increase in abundance of PorA below 50% aerobiosis to a maximum of approximately eightfold higher abundance at 25% aerobiosis compared to 100% and above. Several other outer membrane proteins important in host cell interactions, such as the fibronectin‐binding adhesins CadF (Cj1478) and FlpA (Cj1279), the porin/tyrosine kinase CjkT (Omp50) and the BamA beta‐barrel insertion machine were significantly more abundant at 40% aerobiosis compared to 150% aerobiosis (Table 1 and Supporting Information Table S2). Other proteins important in pathogenicity/host interactions include the MacAB multi‐drug efflux system which was only detected at 40% aerobiosis and the secreted proteases HtrA and Cj0511, which were modestly more abundant at the lower oxygen availability (Table 1). Many proteins encoded in the capsule locus involved in distinct steps of capsular polysaccharide synthesis (CPS), including heptose synthesis (HddA, HddC), sugar transfer or epimerisation reactions (Cj1418, Cj1426, Cj1428, Cj1429, Cj1432, Cj1434, Cj1438, Cj1439, Cj1441) and transport/assembly (KpsD) showed a modest (approximately twofold) but statistically significant increase in abundance at 40% versus 150% aerobiosis (Supporting Information Table S2). In addition, Cj1136, a galactosyltransferase previously shown to be involved in LOS biosynthesis (Javed *et al*., 2012), was 4.3‐fold more abundant at 40% aerobiosis. Interestingly, several enzymes of the N‐linked glycosylation pathway (PglB, PglH, PglK, PglJ, PglA and PglF) showed highly significant fold‐increases ranging from 3.1‐fold (PglF) to 44.5‐fold (PglB), at 40% compared to 150% aerobiosis (note that other Pgl proteins such as PglC, D and L were not detected in our analysis). However, SDS‐PAGE of the same cell‐free extracts as used for the label‐free proteomic analyses followed by blotting with Soybean Agglutinin (SBA) lectin, showed that, overall, N‐linked glycosylation on specific proteins was not more prevalent at 40% compared to 150% aerobiosis (Supporting Information Fig. S6). Further work is therefore required to determine if the increased abundance of these Pgl enzymes has any biological significance.

**Table 1:** Abundance of selected proteins related to colonization, virulence and anti‐microbial resistance at low and high aerobiosis steady‐states.

Category	Protein	Mean emPAI 40% aerobiosis	Mean emPAI 150% aerobiosis	Fold‐change	*P*‐value
Protein glycosylation	PglB (Cj1126)	0.420	0.009	44.5	< 0.0001
	PglH (Cj1129)	0.577	0.047	12.3	0.0029
	PglK (Cj1130)	1.207	0.121	9.9	< 0.0001
	PglJ (Cj1127)	2.142	0.424	5.0	< 0.0001
	PglA (Cj1125)	1.130	0.247	4.6	0.00038
	PglF (Cj 1120)	1.571	0.488	3.2	< 0.0001
	PseE (Cj1337)	0.468	0.130	3.6	0.0140
Outer membrane	CjkT (Cj1170)	5.883	0.878	6.7	< 0.0001
	BamA (Cj0129)	2.735	0.631	4.3	< 0.0001
	CadF (Cj1478)	15.989	6.592	2.4	< 0.0001
	FlpA (Cj1279)	10.570	6.078	1.7	0.0009
Multi‐drug efflux	MacA (Cj0608)	0.310	0	–	0.0038
	MacB (Cj0607)	0.370	0	–	0.0052
	CmeF (Cj1033)	0.629	0.179	3.5	0.0025
Proteases	HtrA (Cj1228)	9.484	4.381	2.2	< 0.0001
	Cj0511	5.868	3.351	1.8	0.0002

The mean emPAI values are derived from three independent steady‐states at each aerobiosis condition. The complete data are given in Supporting Information Table S2.

### Response of specific solute transport systems and catabolic enzymes to increasing aerobiosis

**Figure 5:**
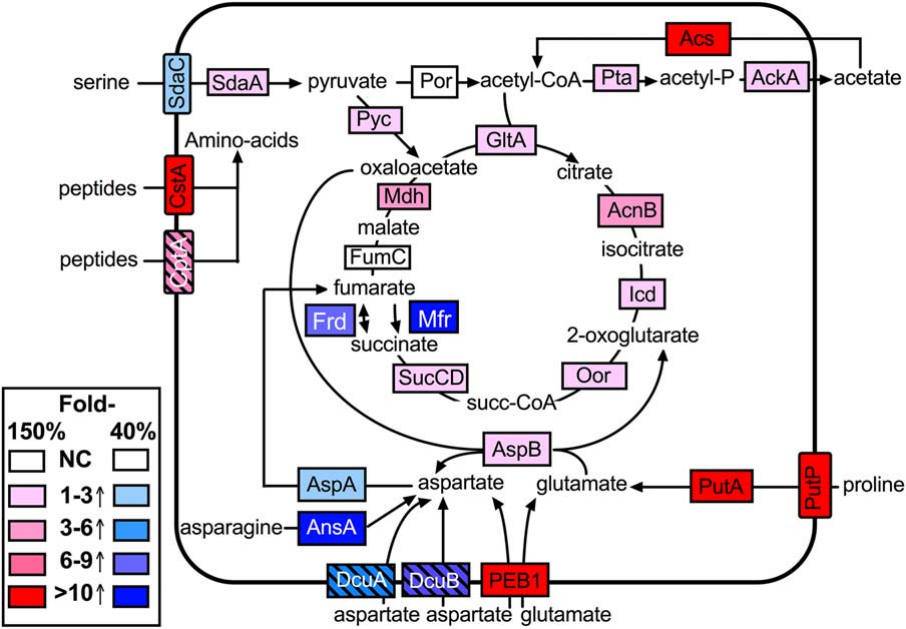
Responses of central catabolic enzymes and associated solute transporters to high and low aerobiosis. The citric‐acid cycle and associated catabolic pathways are depicted within the *C*. *jejuni* cell with selected solute transport systems shown embedded in the cytoplasmic membrane. The fold‐increases in protein abundance at either high (150%) or low (40%) aerobiosis compared to the opposite condition are shown in colour‐coded format based on the LC‐MS/MS data in Supporting Information Table S2. In the case of some low abundance membrane transporters not detected in the proteomics analysis (CptA, DcuA, DcuB; shown as striped boxes), data from the gene expression changes in Supporting Information Table S1 were used to illustrate the pattern of regulation. NC; no significant change.

The transcriptomic and proteomic data provide numerous new insights into how *C*. *jejuni* regulates solute transporters and catabolic pathways as aerobiosis increases (summarised for key pathways in Fig. 5). Several citric‐acid cycle enzymes including citrate synthase (GltA) aconitase (AcnB), isocitrate dehydrogenase (Icd), 2‐oxoglutarate:acceptor oxidoreductase (Oor), succinyl‐CoA synthase (SucCD) and malate dehydrogenase (Mdh) were more abundant at 150% versus 40% aerobiosis (Supporting Information Table S2) and these data are supported by the corresponding gene expression changes (Supporting Information Table S1). The acetyl‐CoA synthetase gene (*acs*, *cj1537*) was downregulated in the high‐to‐low oxygen transition (approximately fourfold after 30 min; Supporting Information Table S1) and the abundance of Acs was 63‐fold higher at 150% compared to 40% aerobiosis (Supporting Information Table S2). Acs abundance was also strongly positively correlated with increasing aerobiosis over the range of steady‐states analysed by 2D‐gels (Supporting Information Table S3).

We found that the abundance of specific peptide and amino acid transporters and their associated catabolic enzymes increased at higher oxygen availability (Fig. 5). *Campylobacter jejuni* can use certain peptides for growth (Rasmussen *et al*., 2013; Vorwerk *et al*., 2014) and the CstA transporter (Cj0917) was identified as allowing the utilisation of a number of di‐ and tri‐peptides (Rasmussen *et al*., 2013). We observed a strong oxygen‐dependent decrease in *cstA* gene expression (∼8.8‐fold after 30 min) in the transition from high to low aerobiosis (Supporting Information Table S1) and CstA was only detected in the label‐free proteomics analysis in the 150% aerobiosis steady‐states (Supporting Information Table S2). The *cptA* peptide transporter gene (Cj0204; Vorwerk *et al*., 2014) was also downregulated approximately threefold after 30 min during the transition (CptA was not detected in the MS analysis). Changes in the individual components of the aspartate/glutamate specific PEB1 ABC‐transporter (Cj0919–922; Leon‐Kempis *et al*., 2006) are shown in Fig. 4D–F. During the high to low aerobiosis transition, the *cj0919c‐922c* genes all transiently decreased in expression by approximately threefold to sevenfold (Supporting Information Table S1; plotted for the *cj0921c/peb1a* and *cj0922c/peb1C* genes in Fig. 4D). The Peb1A (periplasmic binding‐protein) and Peb1C (ATP‐binding cassette protein) proteins were 13‐fold and 16‐fold more abundant respectively in the 150% aerobiosis steady‐states compared to 40% aerobiosis (Fig. 4E) and the 2D‐gel analysis showed a gradual increase in abundance of Peb1A from 25% up to 200% aerobiosis with an approximately sixfold change (Fig. 4F). Although Peb1A has been characterised as an adhesin (Pei *et al*., 1998) as well as an aspartate/glutamate solute binding‐protein (Leon‐Kempis *et al*., 2006), the pattern of regulation of the PEB1 transporter strongly suggests a primary role coupled to catabolism. The aspartate:glutamate aminotransferase AspB (Cj0762) which is essential for growth on glutamate (Guccione *et al*., 2008) was also more abundant at 150% aerobiosis (Supporting Information Table S2 and Fig. 5). However, the aspartase protein AspA (Cj0087), which deaminates aspartate to fumarate was regulated oppositely and in the label‐free analysis was 3.4‐fold more abundant at 40% compared to 150% aerobiosis (Supporting Information Table S2). During the 150–40% aerobiosis transition, *aspA* gene expression was also transiently upregulated (Supporting Information Table S1). AspA has previously been shown by batch culture studies to be upregulated at low oxygen at the transcriptional and protein/enzyme activity levels (Woodall *et al*., 2005; Guccione *et al*., 2008) in the same manner as found in this study. AspA is a key enzyme in aspartate and glutamate catabolism that is important for host colonisation (Guccione *et al*., 2008; Hofreuter *et al*., 2008); its pattern of regulation reflects its role in fumarate provision at low oxygen availability (Fig. 5) to support fumarate respiration. This is correlated with the upregulation of the co‐transcribed *dcuA* C4‐dicarboxylate transport gene and the unlinked *dcuB* gene at low aerobiosis (Supporting Information Table S1 and Fig. 5), which have recently been shown to function in both aspartate and fumarate transport under low oxygen conditions (Wösten *et al*., 2017). Interestingly, the cytoplasmic asparaginase AnsA was also much more abundant at 40% aerobiosis compared to 150% (Supporting Information Table S2 and Fig. 5), consistent with a role in supplying aspartate to AspA at low oxygen.

**Figure 6:**
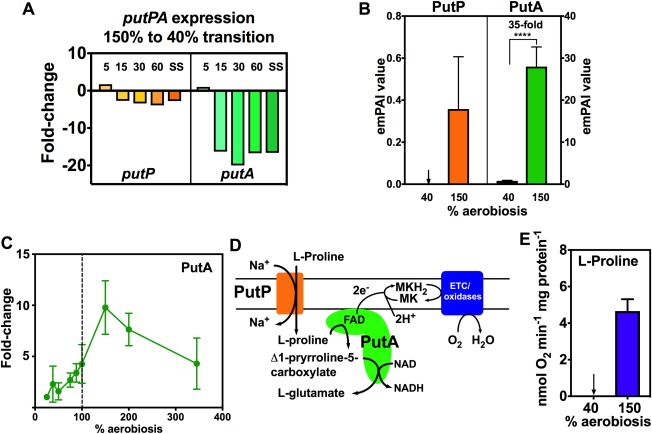
Proline transporter (PutP), proline utilization A (PutA) gene and protein expression and l‐proline oxidation activity are enhanced at high aerobiosis. A. *putP* and *putA* gene expression changes during the 150–40% aerobiosis transition (normalised to onefold at 150% aerobiosis), showing the large decrease in putA expression. B. Relative abundance of PutP and PutA in steady‐states at 40% and 150% aerobiosis, measured by LC‐MS/MS (**** *p* < 0.0001 by Students *t*‐test). C. PutA abundance at steady‐states from 25 to 340% aerobiosis, expressed as the fold‐change in the means of the normalised spot volumes from the 2D‐gel analysis raw data in Supporting Information Table S3. D. Schematic diagram showing how proline transport and oxidation to glutamate are linked to electron transfer via FAD in the proline dehydrogenase domain of PutA to the respiratory chain. E. l‐proline dependent oxygen consumption rates in cells from high and low aerobiosis steady‐states. Where shown, error bars indicate standard deviation of the mean.

The proline utilisation A gene (*putA*) showed the largest fold decrease (∼20‐fold) of any gene during the transition from 150% to 40% aerobiosis (Fig. 6A). The co‐transcribed Na^+^:proline symporter gene *putP* showed a similar pattern but with a smaller fold‐change (Fig. 6A). Correspondingly, in the label‐free proteomic analysis PutA was ∼35‐fold higher in abundance at 150% compared to 40% aerobiosis and PutP was only detected at 150% aerobiosis (Fig. 6B). The 2D‐gel proteomics analysis over a range of oxygen availabilities in steady‐state showed a progressive increase in PutA abundance up to 150% aerobiosis and a decline thereafter (Fig. 6C). PutA oxidises l‐proline to glutamate (Fig. 6D); the electrons from PutA mediated proline oxidation to 5‐pyrroline carboxylate are transferred via the flavin co‐factor in PutA to the menaquinone pool where they can be used for respiration (Fig. 6D). In confirmation of the transcriptomic and proteomic data, we showed that l‐proline dependent oxygen respiration was only detectable in cells grown at 150% aerobiosis (Fig. 6E). Taken together, the data suggest that proline catabolism in *C*. *jejuni* is strongly coupled to respiration with oxygen as the electron acceptor.

### Co‐ordinated cytoplasmic responses to oxidative stress above 100% aerobiosis and correlation with increased abundance of iron‐sulphur cluster biogenesis proteins

**Figure 7:**
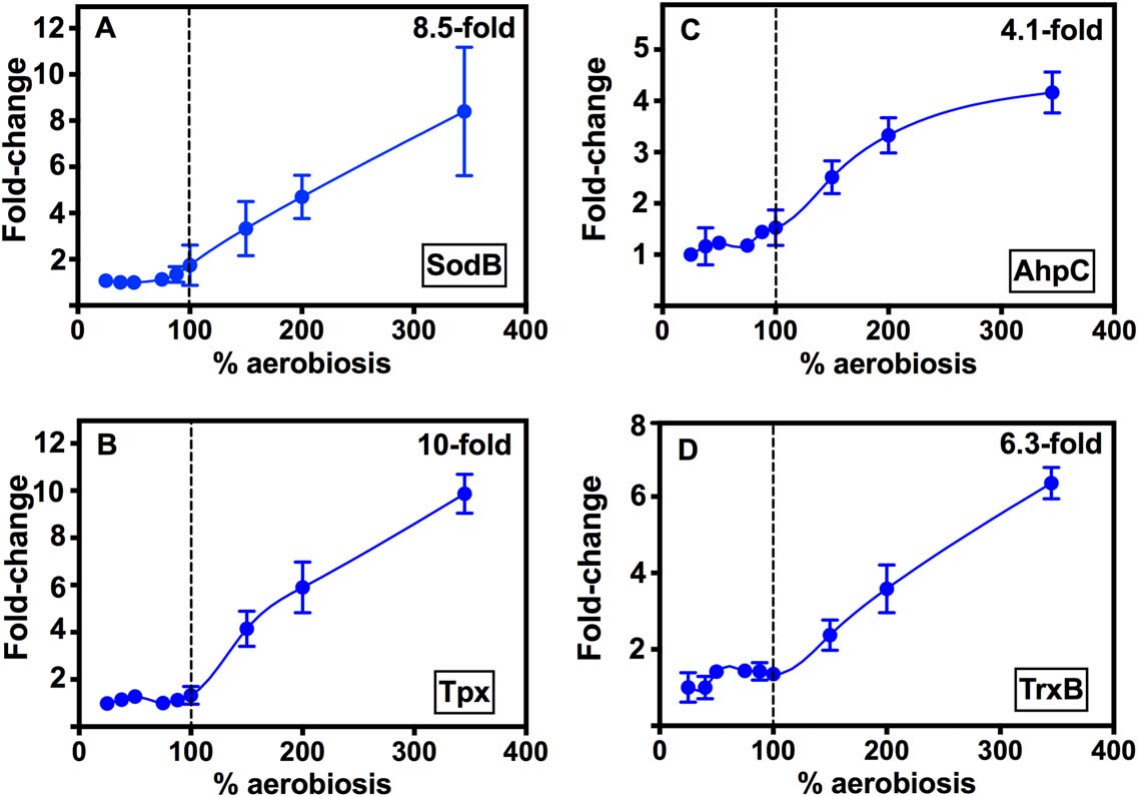
The abundance of oxidative stress protection enzymes increases only above 100% aerobiosis. Protein abundance at each steady‐state is expressed as the fold‐change in the means of the normalised spot volumes from the 2D‐gel analysis raw data in Supporting Information Table S3. (A) SodB (spot 1889), (B) Tpx (spot 1547), (C) AhpC (spot 1512), (D) TrxB (spot 1224). Errors bars show the standard deviation of the mean in each case.

It is well established that exposure of bacterial cells to excess oxygen markedly increases the rate of ROS production, particularly H_2_O_2_ (Seaver and Imlay, 2004), so we were particularly interested in the oxidative stress response of *C*. *jejuni* as the oxygen availability is increased above 100% aerobiosis. At 150% aerobiosis, an initial analysis of proteins with significantly higher emPAI values relative to 40% aerobiosis (Supporting Information Table S2) showed that virtually all of the known enzymes necessary for oxidative stress protection in *C*. *jejuni* were more abundant. These include superoxide dismutase (SodB), the thiol peroxidases Tpx and AhpC and also thioredoxin reductase (TrxB), which supplies reductant via thioredoxin itself to Tpx and probably AhpC. These proteins have well‐established roles in *C*. *jejuni*; SodB converts superoxide into hydrogen peroxide, which is destroyed by a variety of enzymes including catalase and the thiol peroxidases AhpC, Tpx and also Bcp (Atack *et al*., 2008; Flint *et al*., 2016). The putative NADPH:quinone reductase homologue Cj1545 (MdaB) was 26.4‐fold more abundant at 150% compared to 40% aerobiosis; MdaB is known to be involved in oxidative stress protection in other bacteria, for example *Helicobacter pylori* (Wang and Maier, 2004) but its role in *C*. *jejuni* is unclear (Flint *et al*., 2016). The 2D‐gel based proteomic analysis confirmed the above results and showed that while all of the above enzymes are remarkably constant between 25 and 100% aerobiosis, they become progressively more abundant above 100% aerobiosis (Supporting Information Table S3 and Fig. 7). This is clearly illustrated in Fig. 7 for SodB (maximum 8.5‐fold change between steady‐states), Tpx (10‐fold change), AhpC (fourfold change) and TrxB (6.3‐fold change), where it is strikingly apparent that there is a threshold at 100% aerobiosis, indicating enzyme synthesis is only increased above a fairly constant basal level when oxygen becomes in excess of that required for fully aerobic catabolism and where ROS would start to accumulate.

**Figure 8:**
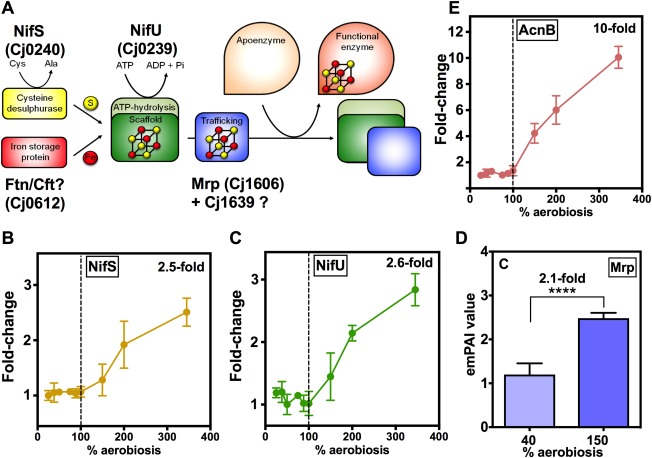
Proteins involved in the biogenesis of Fe‐S clusters become more abundant above 100% aerobiosis. (A) Schematic diagram of the assembly pathway for Fe‐S clusters, involving the key proteins NifS, NifU and Mrp. The source of iron is not clear, but may originate from the ferritin Ftn/Cft. Cj1639 may also play a role in the pathway (Kendall *et al*., 2014). The change in abundance of NifS (B) and NifU (C) at each steady‐state is expressed as the fold‐change in the means of the normalised spot volumes from the 2D‐gel analysis (raw data in Supporting Information Table S3). For Mrp, which was not detected on the 2D‐gels, (D) shows the emPAI values from the LC‐MS/MS analysis at 40% and 150% aerobiosis. (**** *p* < 0.0001 by Students *t*‐test). In (E), the abundance of the Fe‐S enzyme aconitase (AcnB) at each steady‐state is expressed as the fold‐change in the means of the normalised spot volumes from the 2D‐gel analysis (raw data in Supporting Information Table S3). Error bars represent standard deviation of the mean.

Significantly, we noted from the 2D‐gel analysis that two key proteins which are responsible for the biogenesis of iron‐sulphur clusters (Fig. 8A), the cysteine desulfurase NifS (Cj0239) and the Fe‐S scaffold/assembly protein NifU (Cj0240) also show a clear pattern of increasing abundance only above 100% aerobiosis (Fig. 8B and C respectively). In addition, the Fe‐S cluster trafficking protein Mrp (Cj1606) was twofold more abundant at 150% compared to 40% aerobiosis (Fig. 8D). In previous work, we showed that *C*. *jejuni* possesses a number of Fe‐S cluster enzymes that are vulnerable to oxidative damage (Kendall *et al*., 2014). These include the l‐serine dehydratase SdaA and the citric‐acid cycle enzymes POR, OOR, aconitase and fumarase. Of these, the proteomic data in Supporting Information Tables S2 and S3 show that aconitase shows a parallel pattern of increasing abundance at > 100% aerobiosis (plotted in Fig. 8E). SdaA and fumarase were not detected in the 2D‐gel analysis, but the OorA and OorB subunits followed the same general trend and 2‐oxoglutarate dependent respiratory activity was approximately threefold increased at 150% compared to 40% aerobiosis (Supporting Information Fig. S2D). In contrast, the abundance of some of the other non‐FeS cluster (i.e., oxygen stable) citric‐acid cycle enzymes, for example succinyl‐CoA synthase (SucC) and NAD‐dependent malate dehydrogenase (Mdh) showed a different pattern, with an increase occurring between 25% to at least 100% aerobiosis but stabilising thereafter (Supporting Information Table S3). There is thus a correlation between the changes seen in some key Fe‐S cluster containing enzymes and the Fe‐S biogenesis system under conditions where oxidative stress is clearly increasing (Figs 7 and 8). Fe‐S cluster repair in *C*. *jejuni* is thought to be inefficient but the hemerythrin Cj0241 may play a role in protection (Kendall *et al*., 2014). Cj0241 was not detected in our proteomic analysis but *cj0241c* gene expression decreased in the transition from high to low oxygen availability (Supporting Information Table S1), consistent with a role at high aerobiosis.

*Campylobacter jejuni* encodes two periplasmic di‐haem cytochrome *c* peroxidases (Cj0358 and Cj0020 in NCTC11168). Interestingly, Cj0358 was threefold more abundant at 40% aerobiosis compared to 150% while Cj0020 showed no statistical change (Supporting Information Table S2). *cj0358* gene expression also increased during the transition to lower aerobiosis, while *cj0020c* expression was unchanged (Fig. 3), consistent with the proteomic data. This pattern is the opposite to that of the cytoplasmic oxidative stress related proteins shown in Fig. 7 and suggests a distinct role for Cj0358 in the periplasm at low oxygen availability.

## Discussion

The chemostat approach used in this study has allowed us, for the first time, to compare gene expression and protein synthesis in *C*. *jejuni* cells undergoing fully aerobic catabolism with those cells where oxygen availability is significantly reduced, at the same growth rate. We were able to establish stable carbon‐limited steady‐states in a minimal medium across a wide range of oxygen inputs and we have demonstrated that in *C*. *jejuni*, as in *E*. *coli* (Alexeeva *et al*., 2002), there is a linear relationship between rate of acetate excretion and the oxygen input, allowing an aerobiosis scale to be devised. The virtue of this scale is that it is independent of individual chemostat geometry, culture volume and gas transfer characteristics and can thus be used to compare results from different laboratories (Alexeeva *et al*., 2002). Although *C*. *jejuni* cannot grow under full aeration conditions with atmospheric oxygen levels, our lab strain of NCTC11168 was quite oxygen tolerant and could be grown in steady‐state at up to 345% aerobiosis. Indeed, a previous study comparing the characteristics of the original isolated NCTC11168 strain with a lab‐passaged strain found many differences, including an increased oxygen tolerance (Gaynor *et al*., 2004). Below 40% aerobiosis, the cultures became increasingly oxygen‐ rather than carbon‐limited and this resulted in a decreased molar growth yield and detectable l‐serine in the culture supernatants.

The transcriptomic analysis we report here has allowed a comparison between steady‐states at high and low aerobiosis and also insight into the temporal changes in gene expression accompanying a high‐to‐low aerobiosis shift, as would occur during host colonization from the environment. As expected, and as also observed in batch cultures by van der Stel *et al*. (2017), major changes were found to occur in the expression of genes encoding alternative electron transport/respiratory chain components, as the cells adapted to a lowered oxygen availability. Most obvious was the up‐regulation of hydrogenase genes and of the genes for the terminal reductases for nitrate, nitrite, TMAO and fumarate. The apparent lack of regulation of the formate dehydrogenase genes in our study was due to the absence of selenium in our standard chemostat medium, which is known to be essential for expression of this enzyme via an effect on mRNA stability, as well as the activity of the enzyme itself (Shaw *et al*., 2012).

Some of the upregulated genes shown in Fig. 2 are known to be under the control of the oxygen‐sensing transcription factor FNR in other bacterial species (Crack *et al*., 2012). However, the identities of the sensor[s]/transcription factor[s] mediating oxygen regulation in *C*. *jejuni*, which lacks both FNR and ArcA homologues, are unknown. At least three regulatory systems are, however, known to affect expression of different electron transport genes in *C*. *jejuni*. These are the RacRS two‐component system (van der Stel *et al*., 2015), the Cj1491/Cj1492 two‐component system (Luethy *et al*., 2015) and the LysR family regulator Cj1000 (Dufour *et al*., 2013). Of these, RacRS is understood in most detail and responds to low oxygen in the presence of nitrate or TMAO to effectively repress fumarate transport and respiration genes (van der Stel *et al*., 2015). However, although there is no evidence so far to indicate that RacS is an oxygen‐sensor *per se*, it might be a redox sensor as it depends for activity on an oxidation sensitive periplasmic disulphide bond (van Mourik, 2011). We showed by modelling that RacR activity decreases during the high to low oxygen shift and this can account for changes in expression of its major target genes, i.e., *mfr, aspA, dcuA, cj0358* seen in this study. The signal for the Cj1491/Cj1492 system is unknown but this system seems mainly to regulate the gluconate dehydrogenase (*cj0414/415*) and possibly the *c*‐type cytochrome *cj0037c* (*cccC*) genes (Luethy *et al*., 2015), both of which we identified as oxygen‐regulated in this study. The Cj1492 response regulator was also approximately twofold higher in abundance at 40% versus 150% aerobiosis (Supporting Information Table S2). The Cj1000 regulator seems to have quite small and probably indirect regulatory effects on several electron transport related genes (*frd, mfr, hyd, nrf, sor* and *aspA, dcuA*; Dufour *et al*., 2013). Thus, as yet unknown regulators are responsible for the oxygen‐regulation of *nap, nrf*, *tor* and many other genes demonstrated here.

Recently, it has been shown that short‐chain fatty acids like acetate can influence metabolic gene expression in *C*. *jejuni* (Luethy *et al*., 2017). Mutants in *pta/ackA* show reduced expression of a variety of such genes, including *mfr, frd, sdaA and peb1a/b/c*. For some of these genes, it was shown that expression could be restored upon addition of exogenous acetate (Luethy *et al*., 2017). We were concerned that the inevitable accumulation of acetate in the low aerobiosis cultures in our work might compromise our interpretation that it is low oxygen availability that causes the upregulation of specific genes. However, in the work of Luethy *et al*., (2017) 50–100 mM acetate was required to restore gene expression in the *pta/ackA* mutant (which still produced some acetate); 10 mM exogenous acetate had no effect. In our work, the maximum concentration of acetate we recorded at any steady‐state was about 5 mM. Thus, this low level of acetate accumulation cannot account for the transcriptional patterns we have observed.

The abundances of many proteins correlated well with the transcriptomic data. The label‐free LC‐MS/MS methodology comparing high (150%) and low (40%) aerobiosis gave a deep proteome coverage, which was complemented by the analysis of additional steady‐states using a 2D‐gel and spot‐picking/MALDI‐TOF approach. Overall, the results from the two workflows were in broad agreement. However, some proteins known to be essential for viability were only detected by the label free proteomic analysis in one or other of the two oxygen regimes analysed. These included MurF, MsbA, PlsY and Lnt involved in cell wall and lipid metabolism, which were detected at 40% but not 150% aerobiosis. These are membrane‐associated proteins that may not be extracted consistently at low abundance. Indeed, membrane proteins in particular are over‐represented in the list of proteins that were undetected in either steady‐state condition (Supporting Information Table S2). Some zero emPAI values in Supporting Information Table S2 may thus represent proteins that are very low in abundance; for example, MfrA and MfrB. These were, however, detected in the cells from 150% aerobiosis steady‐states when analysed by 2D‐gels (Supporting Information Table S3) but at very low abundances consistent with their oxygen regulation.

From an analysis of the proteomic data at levels of aerobiosis below 100%, we obtained evidence for increased abundance of a variety of proteins that are associated with colonisation and virulence, particularly periplasmic and cell envelope associated proteins. It seems likely, therefore, that along with short‐chain fatty acids (Luethy *et al*., 2017), the reduced oxygen availability that *C*. *jejuni* encounters during colonisation of the host intestine may be used as a signal to increase expression of certain proteins needed for growth *in vivo*. One of the clearest examples concerned the major outer membrane porin (MOMP; PorA), which markedly increased in abundance at steady‐states below 50% aerobiosis; the *porA* gene was also upregulated transiently in the high to low transition experiment. The MOMP has many important roles in host cell interaction in *C*. *jejuni* (e.g., Moser *et al*., 1997; Mahdavi *et al*., 2014). A previous proteomic study of *C*. *jejuni* invading cultured cells also found evidence of high MOMP abundance at low oxygen conditions (Liu *et al*., 2012), although another study using 2D‐gels with ‘oxygen‐acclimated cells’ found higher PorA and CadF at high oxygen conditions (Sulaeman *et al*., 2012). However, the latter study used plate grown cells (i.e., colonies) of strain 81–176 on complex media for comparisons under different oxygen atmospheres generated in gas jars, very different from the experimental approach employed here. It is possible that the cells in those colonies were in fact oxygen‐limited. As discussed above, we believe it is essential to use well‐defined oxygen regimes with homogenous exponentially growing cells at the same growth rate in order to make confident conclusions about oxygen‐regulated proteins. Interestingly, in our work the behaviour of three adhesins, CadF, FlpA and Peb1A was different with respect to oxygen, with CadF and FlpA increased in abundance at 40% aerobiosis, while the Peb1A protein was reduced. A more detailed analysis of gene and protein expression of the components of the PEB1 system (Fig. 4) clearly showed a positive effect of oxygen. We interpret this as supporting the conclusion that the primary role of Peb1A is as an essential part of the PEB1 ABC‐transporter, an uptake system for aspartate and glutamate for catabolic purposes, whereas CadF and FlpA are dedicated adhesins.

The strong oxygen‐dependent pattern of *putAP* gene expression, abundance of PutA and PutP proteins as well as l‐proline oxidation activity is very striking. In common with many bacteria, the *C*. *jejuni* PutA protein is a bifunctional enzyme, oxidizing l‐proline to pyrroline‐5‐carboxylate (P5C) in the N‐terminal FAD‐containing proline dehydrogenase domain, transferring the electrons to menaquinone in the membrane and then further oxidising the glutamate semialdehyde formed spontaneously from P5C to glutamate via the C‐terminal NAD‐dependent P5C dehydrogenase domain (Fig. 6D). Some PutA proteins are tri‐functional; they have a short additional N‐terminal DNA‐binding domain that controls expression of *putA* and *putP* in response to proline availability (Zhou *et al*., 2008). Interestingly, a study of the regulation of *putA* in *Salmonella* showed that *putA* expression depends on oxic conditions and that the effect of oxygen was mediated by PutA itself, through its DNA binding activity (Maloy and Roth, 1983). However, the *C*. *jejuni* PutA does not have a DNA‐binding domain and so the *putPA* operon must be regulated by an unidentified transcription factor, perhaps analogous to the Lrp‐like PutR activator found in some bacteria (Lee and Choi, 2006), but further work will clearly be needed to define the mechanism of regulation.

Our results also showed a very clear pattern of a progressive increase in the abundance of a range of cytoplasmic enzymes mediating oxidative stress resistance, but only above 100% aerobiosis. This pattern suggests that ROS production at aerobiosis levels above that needed for complete substrate oxidation is actively countered by up‐regulating synthesis of several detoxification enzymes. Thus, systems for both superoxide (SodB) and peroxide (Tpx, Bcp and AhpC thiol peroxidases) detoxification followed the same pattern as did the thioredoxin system (Trx and TrxB) that supplies the reductant necessary for the thiol peroxidases. Note that for unknown reasons we did not detect catalase in our proteomics work. The cellular molar growth yield starts to decrease above 150% aerobiosis (Fig. 1C) and we were unable to establish a steady‐state above 345% aerobiosis. Thus, although this concerted response is partially effective, the data do suggest fatal oxidative damage to vulnerable targets is occurring above 150% aerobiosis. Key Fe‐S enzymes are known to be inactivated by excess oxygen in *C*. *jejuni* (Kendall *et al*., 2014) and significant increases above 100% aerobiosis were observed here for NifS, NifU and Mrp which are the cysteine desulfurase, scaffold and trafficking proteins, respectively, needed for Fe‐S cluster biogenesis (Py and Barras, 2010; Kendall *et al*., 2014). An increase in the abundance of the Fe‐S enzymes themselves would also help to ensure activity is maintained despite a fraction of the proteins becoming inactivated. This was most clearly observed in the case of aconitase (AcnB; Fig. 8). Taken together, the proteomic data clearly indicate that the cells are not only responding to increased oxidative stress above 100% aerobiosis by increasing ROS defence enzyme synthesis but also increasing their capacity for Fe‐S cluster biogenesis. Increases in both oxidative stress genes and Fe‐S cluster biogenesis genes have also been observed in *E*. *coli* cells undergoing an anoxic‐oxic shift (Partridge *et al*., 2006).

Finally, unlike the cytoplasmic peroxidatic enzymes discussed above, the regulatory behaviour of the two periplasmic cytochrome *c* peroxidases (CCPs) in *C*. *jejuni* is clearly distinct, with *cj0358* gene expression and Cj0358 protein abundance being increased at low aerobiosis but *cj0020c* unchanged. Most bacteria possess a single CCP, but *C*. *jejuni* strains are unusual in having two phylogenetically distinct enzymes. Cj0358 (CcpA2) is a typical di‐haem CCP like the well‐studied enzyme from *P*. *aeruginosa* (Atack and Kelly, 2007). However, Cj0020 (DocA or CcpA1) is not closely related to Cj0358, but is more similar to the tri‐haem quinol peroxidase enzymes from *Actinobacillus/Aggregatibacter* (Atack and Kelly, 2007). Mutant studies (Hendrixson and DiRita, 2004) indicate that Cj0020 plays a role in chicken colonization but other studies do not suggest that either Cj0020 or Cj0358 play a major role in oxidative stress protection (Bingham‐Ramos and Hendrixson, 2008; Flint *et al*., 2014, 2016). The regulatory pattern observed with Cj0358 is in keeping with work on most other bacterial CCPs, which are upregulated under microaerobic or anaerobic growth conditions (van Spanning *et al*., 1997). In *C*. *jejuni*, *cj0358* is a member of the RacR regulon (van der Stel *et al*., 2015) and our modelling showed that RacR contributes to the regulatory pattern of *cj0358*. The actual physiological role of bacterial CCPs has long been unclear (reviewed in Atack and Kelly, 2007). However, Khademian and Imlay (2017) have recently proposed from regulatory and functional studies in *E*. *coli* that hydrogen peroxide can act as a true electron acceptor via CCP activity. Hydrogen peroxide might be generated within the periplasm under low oxygen conditions as a side reaction of periplasmic formate oxidation by formate dehydrogenase, as previously shown for *Campylobacter mucosalis* (Goodhew *et al*., 1988).

In conclusion, we have shown that under the microaerobic conditions (< 100% aerobiosis) that prevail in the host caecum and intestinal mucosa, *C*. *jejuni* can respond by upregulating (i) proteins involved in hydrogen and formate oxidation and alternative electron transport pathways and (ii) proteins necessary for colonisation and interaction with the host: The predominant proteomic ‘signature’ that characterises aerobic conditions (> 100% aerobiosis) comprises (i) enzymes involved in oxidative stress protection and Fe‐S cluster biogenesis and (ii) enzymes and transporters of both central and some peripheral catabolic pathways that are physiologically linked to oxygen respiration. This study has uncovered the hitherto unrecognised extent to which oxygen affects gene expression in *C*. *jejuni*; how oxygen is actually sensed and how that signal is transduced are key questions for future studies.

## Experimental procedures

### Bacterial strains, media and batch culture conditions

For routine growth*, C*. *jejuni* strain NCTC 11168 was cultured at 37°C under reduced oxygen and enhanced carbon dioxide gas atmosphere conditions (10% v/v O_2_, 5% v/v CO_2_ and 85% v/v N_2_) in a MACS‐VA500 incubator (Don Whitley Scientific Ltd, UK) on Columbia agar (CA) containing 5% v/v lysed horse blood and 10 mg ml^−1^ each of amphotericin B and vancomycin. Liquid cultures were grown in Müller‐Hinton (MH) broth at 37°C, under the gas concentrations above with 50 ml of medium contained in 250 ml conical flasks, mixed by continuous orbital shaking at 120 rpm.

### Continuous chemostat culture

Cells were grown in a carbon (serine)‐limited chemostat (Infors HT Labfors 3 monitored and controlled using Infors Iris 5 software; Infors, Switzerland) in the defined medium described previously (Guccione *et al*., 2010), based on MEM‐alpha medium. The required input gas composition was obtained by proportional mixing from a compressed air line plus a 90:10% v/v nitrogen/CO_2_ gas cylinder. The input CO_2_ concentration varied from ∼2% v/v to 10% v/v over the range of oxygen inputs used. Based on our previous work (Al‐Haideri *et al*., 2016), 2% v/v CO_2_ is in excess of the cells growth requirements. The culture volume was 885 ml, the temperature was maintained at 37°C by a thermostatic water jacket, the gas sparging rate was 0.5 l min^−1^ with a stirring rate of 350 rpm and the pH was maintained at 7 ± 0.1 with automatic addition of 1 M NaOH or H_2_SO_4_. The vessel was inoculated aseptically to an OD_600_ of 0.1 with cells grown in MH batch culture under standard microaerobic conditions. After inoculation, cells were initially grown as a batch culture for 6 h, reaching an OD600 of ∼0.6; at this point fresh media was fed into the vessel at a dilution rate of 0.2 h^−1^ until steady‐state was reached, defined by a stable optical density for five vessel volumes of fresh media supplied to the chemostat. Each steady‐state was derived from an independent initial batch culture. Samples were taken from the steady‐states for physiological and proteomic analyses as described below. For the high to low oxygen temporal transition experiment, cultures initially grown to steady state with a gas mixture providing 150% perceived aerobiosis (7.5% v/v O_2_, 6.4% v/v CO_2_ & 85.7% v/v N_2_) were switched to 40% aerobiosis (1.88% v/v O_2_, 9.11% v/v CO_2_ & 88.9% v/v N_2_) at time zero. Samples were then taken at the time points indicated below and when the culture reached the new steady state and processed for transcriptomic analyses.

### ^1^H nuclear magnetic resonance spectroscopy

Chemostat culture samples were centrifuged to remove cells (13,800 × *g*, 5 min) and the supernatants used directly for NMR analysis. ^1^H‐NMR was carried out using a Bruker DRX500 spectrometer operating at 500 MHz, as described by Leon‐Kempis *et al*. (2006). Spectra were acquired into 4096 complex points over a spectral width of 12.5 kHz and the solvent (H_2_O) signal reduced by pre‐saturation for 2 s. Samples (0.45 ml supernatant plus 0.05 ml of D_2_O) were run in 5 mm diameter tubes at 25°C. Chemical shifts and metabolite concentrations were established by reference to 1 mM trimethylsilylpropionate (TSP; 0 ppm) added to all samples. For quantification of peak area, integration was performed using FELIX (Accelrys, San Diego, CA), using the CH and CH_2_ integrals for l‐serine, and CH_3_ integrals for pyruvate and acetate.

### Physiological analyses

Chemostat samples (5 ml) were taken at all steady‐states and time points during the temporal transition experiment and used to determine OD_600_, cell viability and acetate concentration of the supernatant. Cell viability was measured by serial dilution and colony counts on blood agar plates. For acetate determinations, 1 ml of culture supernatant was analysed using the K‐ACET acetic acid analysis kit (Megazyme). Triplicate 10 ml samples were taken for dry weight analysis. Samples were centrifuged (10,000 × g, 10 min), the supernatant discarded, cell pellets washed with distilled water and dried in pre‐weighed metal caps in a hot air oven (105°C). Caps were cooled in a dessicator and weighed on an analytical balance.

### Transcriptomic analysis

Chemostat culture samples (10 ml) for transcriptomic analysis were taken at both 150% and 40% aerobiosis steady‐states and at 5, 15, 30 and 60 min time points after the transition from 150% to 40% aerobiosis by harvesting directly into a mix of 62.5 µl prechilled phenol made up in 1.2 ml of 100% ethanol (to stabilize the RNA). Samples were then centrifuged (8000 × *g* for 4 min, 4°C). Total RNA was purified from cell pellets using an RNeasy Mini kit (Qiagen) with on‐column DNase I treatment as recommended by the suppliers. RNA (10 µg) from experimental samples was used to prepare Cy5‐dUTP labelled cDNA with Superscript III reverse transcriptase (Invitrogen) as recommended by the manufacturer. Genomic DNA (100 ng) for Type II microarray analysis was Cy3‐dUTP labelled with the Klenow fragment (Invitrogen). cDNA and gDNA was mixed and purified using a PCR purification kit (Qiagen), and concentrated in an Eppendorf Concentrator 5301 to a volume of 5 µl. Hybridization buffer (100 µl; Ocimum Biosolutions) was then added to the cDNA/gDNA mix and heated to 95°C for 3 min. This mix was hybridized to a *C*. *jejuni* NCTC 11168 OciChip (Ocimum Biosolutions) for 16 h at 42°C. Slides were washed with 2x SSC + SDS buffer (300 mM NaCl, 30 mM sodium citrate + 0.04% SDS), 1 × SSC and 0.2 × SSC and dried via centrifugation. Slides were scanned with an Affymetrix 428 scanner with data analysis performed using Imagene, version 5.1 and Genesight version 4. Experiments were performed with at least three biological replicates; dye swap experiments were not performed as all cDNA samples were labelled with Cy5‐dUTP and all genomic DNA was labelled with Cy3‐dUTP. The mean values for each channel were log_10_‐transformed and normalized per spot, dividing by control channel, per chip to the 50th percentile. Normalized values were used to calculate the ratio of each experiment to the time 0 (150% aerobiosis) sample. Data was processed using Genespring 7.3.1. Genes exhibiting greater than twofold change in abundance at one or more of the time points with a *p* value of ≤ 0.05 were deemed to be differentially regulated. The Benjamini and Hochberg test was used to correct for false positives.

### Reverse transcription PCR

The same steady‐state samples as used for the microarray analysis were also used for RT‐PCR of five selected genes that showed up or downregulation. Procedures for RNA isolation, purification and analysis by RT‐PCR using the *gyrA* gene for normalisation, followed the standard methods described in our previous work (Al‐Haideri *et al*., 2016). The primers used are shown in Supporting Information Table S1.

### Modelling of RacR activity using TFINFER

Modelling used the program described in Asif *et al*. (2010) derived from the theoretical treatment described by Sanguinetti *et al*. (2006). TFINFER employs a linear approximation in log space to the dynamics of transcription, based on a state space model of the following form:

$$y_{n}\left(t\right)\;=\sum_{m=1}^{q}X_{nm}b_{nm}c_{m}\left(t\right)\;+\;\mu_{n}+\;{ɛ}_{nt}$$

$$c_{m}\left(t\right)\;=\gamma_{m}c_{m}\left(t-1\right)\;+\eta_{mt}$$

Where *y_n_*(*t*) is the mRNA log expression level for gene *n* at time *t*, *X* is a binary connectivity matrix that describes whether gene *n* is bound by transcription factor (TF) *m*, *b_nm_* encodes the regulatory strength with which TF *m* affects gene *n*, and *c_m_*(*t*) is the (log) concentration of active TF *m* at time *t*. The other terms are used to model noise and biases. The model places Gaussian prior distributions over the concentrations *c_m_*(*t*) and strengths *b_nm_* and uses a factorized variational approximation to infer posterior distributions using the microarray time course data (Sanguinetti *et al*., 2006). The connectivity matrix for RacR was constructed from the data in van der Stel *et al*. (2015).

### 2D‐gel electrophoresis, protein identification and quantitation

Chemostat culture samples (15 ml) were taken for proteomic analyses at all steady states. Samples were centrifuged at 4°C (10,000 × *g*, 10 min) and the supernatant removed. The pellet was washed with 50mM Tris HCl pH 8 and then stored at −80°C until processed for analysis. Methods for the analysis of *C*. *jejuni* proteins on 2D‐gels closely followed those described in Holmes *et al*. (2005) and Leon‐Kempis *et al*. (2006). For the first dimension, 200 μg total sample protein was mixed with IPG rehydration buffer (7M urea, 2M thiourea, 2% w/v CHAPS, 18 mM dithiothreitol [DTT], bromophenol blue and 2% pH3–10 NL non‐linear IPG buffer; final volume 370 μl), before loading onto 18 cm 3–10NL Immobiline DryStrips (Amersham Biosciences, UK). Following overnight rehydration, IEF was performed for 80 kVh at 20°C over 24 h using the pHaser system (Genomic Solutions, UK). The focussed strips were treated as described previously Leon‐Kempis *et al*. (2006). Second dimension 10% duracryl gels (28 × 23 cm, 1 mm thick) were prepared for use in the Investigator second Dimension Running System (Genomic Solutions, UK), with electrophoresis at 500 V or 20 W per gel. Proteins were stained by Sypro‐Ruby (Bio‐Rad, UK), destained, and the gels imaged using the Pharos FX+ Molecular Imager with Quantity One imaging software (BioRad, UK). A 532‐nm laser was used for excitation, along with a 605‐nm band‐pass emission filter. The gels were scanned at a 100 μm resolution to produce a 16‐bit image. The laser strength was adjusted for each image to give maximum signal without saturation based on the strongest spot. At least two replicate gels were run with a minimum of one steady‐state per oxygen input condition (50% and 75% aerobiosis) or with two or more independent steady‐states (25, 40, 88, 100, 150, 200 and 345% aerobiosis). Gel images were compared using ProteomWeaver analysis software (Definiens, UK). Background subtraction and normalisation were performed automatically. Protein spots were excised from the gel using the ProPick excision robot (Genomic Solutions, UK) and in‐gel tryptic digestion performed as described by Holmes *et al*. (2005). Tryptic digests were analysed using a Reflex III MALDI‐TOF instrument (Bruker, UK). Proteins were identified by the Protein Mass Fingerprint technique using the MASCOT search tool (Version 2.4.1, Matrix Science; http://matrixscience.com). Protein hits with a MASCOT score greater than 55 (*p* < 0.05) were taken as confirmed identifications.

### Label‐free proteomics by LC‐MS/MS

Cell‐free extracts prepared from three independent steady‐states at both 40% aerobiosis (coded LF_1, LF_2, LF_3) and 150% aerobiosis (coded LF_4, LF_5, LF_6) were separated on 1D SDS‐PAGE gels which were stained with Coomassie blue. Three replicate gel lanes were run for each independent steady‐state sample (coded LF_1A, LF_1B, LF_1C, LF_2A, etc.). The gel lanes were cut up into 1mm squares which were placed in Eppendorf tubes and immersed in miliq water at 4°C overnight, then equilibrated in 200 mM ammonium bicarbonate/50% v/v acetonitrile by three successive incubations in fresh solution for 15 min each at room temperature. The final solution was removed and the gel slices washed twice with acetonitrile and air dried. To each tube, 1 ml of 10 mM DTT in 50 mM ammonium bicarbonate was added and incubated for 30 min at 60°C. The solution was then replaced with 1 ml 100 mM iodoacetamide in 50 mM ammonium bicarbonate and incubated for 30 min at room temperature in the dark. The solution was removed and the gel pieces were equilibrated again with two successive 15 min incubations in 200 mM ammonium bicarbonate/50% v/v acetonitrile, then washed twice with 1 ml acetonitrile and air dried. To each tube of gel pieces, 40 μl of trypsin solution containing 10 ng μl^−1^ of Trypsin Gold (Promega V528A) in 10 mM ammonium bicarbonate was added, followed by a further 80 μl of 10 mM ammonium bicarbonate to ensure all gel pieces were immersed. After incubation overnight at 37°C, an equal volume of 2% (v/v) formic acid was added, the digest was frozen on dry‐ice for 30 min, then thawed and the solution removed to a fresh tube. The remaining gel pieces were incubated for 10 min with 240 μl 50% v/v acetonitrile to recover more digested peptides from the gel; this extract was then added to the first trypsin digest sample. The combined extracted digest samples were then dried down at the Low Drying setting (no heat) on a Speed Vac SC110 (Savant) fitted with a Refrigerated Condensation Trap and a Vac V‐500 (Buchi). The samples were then frozen on dry ice and stored at −80°C until ready for Orbitrap analysis. Triplicate 10 μl injections for each gel lane sample (coded LF_1A_1, LF_1A_2, LF_1A_3, etc.) were made on an LTQ‐Orbitrap mass spectrometer (ThermoElectron) coupled to a nanoflow‐HPLC system (nanoACQUITY; Waters), with parameters as described by Shaw *et al*. (2012). Tandem mass spectra were analysed using MASCOT (version 2.4.1). Mascot was searched with a fragment ion mass tolerance of 0.50 Da and a parent ion tolerance of 5.0 ppm. Carbamidomethyl of cysteine was specified in Mascot as a fixed modification. Oxidation of methionine was specified in Mascot as a variable modification. Scaffold (version 4.6.1, Proteome software Inc., Portland, USA) was used to validate MS/MS based peptide and protein identifications. Peptide identifications were accepted if they could be established at greater than 99.9% probability by the Scaffold Local FDR algorithm. Protein identifications were accepted if they could be established at greater than 99.9% probability and contained at least two identified peptides. Protein probabilities were assigned by the Protein Prophet algorithm (Nesvizhskii *et al*., 2003). Proteins that contained similar peptides and could not be differentiated based on MS/MS analysis alone were grouped to satisfy the principles of parsimony. Proteins were annotated with standard GO terms from NCBI. The emPAI values (Ishihama *et al*., 2005) calculated by the Scaffold program were used for comparing the relative abundance of proteins at 40% and 150% aerobiosis.

### Detection of glycosylated proteins and c‐type cytochromes on gels

Glycosylated proteins were detected on blots of SDS‐PAGE gels following reaction with soybean agglutinin lectin conjugated with horse‐radish peroxidase (SBA‐HRP) Cell‐free extracts prepared by sonication were run on 12.5% SDS‐PAGE gels and electroblotted onto nitrocellulose membrane (Hybond‐C extra, GE Healthcare UK). The membrane was washed three times in phosphate‐buffered saline plus 0.05% v/v Tween‐20 (PBST) followed by incubation with PBST plus SBA‐HRP (1 μg ml^−1^ final concentration) for 1 h. After washing the membrane three times in PBST, glycosylated proteins were visualised by enhanced chemiluminesence (ECL kit; GE Healthcare, UK) and CCD camera imaging. For the detection of *c*‐type cytochromes on gels, the method described in Liu and Kelly (2015) was adopted, where the covalent attachment of the haem moiety was preserved in cell‐free extract samples by gentle denaturation for 1 h at 42°C in Lamemli SDS sample buffer without mercaptoethanol. After electrophoresis by Tricine‐SDS‐PAGE and electroblotting onto nitrocellulose membrane as above, bound haem was detected as haem‐associated peroxidase activity using enhanced chemiluminesence with visualisation by CCD imaging as for glycosylation blots.

### Substrate oxidation and enzyme assays

Oxygen electrode assays were performed as described previously (Liu and Kelly, 2015). The activity of MfrA was measured as the fumarate dependent oxidation of reduced methyl‐viologen as described by Guccione *et al*. (2010) in periplasmic extracts prepared by osmotic shock from cells directly harvested from the chemostat.

### Data availability and accession numbers

The raw microarray data has been submitted to ArrayExpress with the accession number E‐MTAB‐5743. The label‐free mass spectrometry proteomics data have been deposited to the ProteomeXchange Consortium via the PRIDE partner repository with the dataset identifier PXD006467 and DOI 10.6019/PXD006467.

## Supporting information

Additional Supporting Information may be found in the online version of this article at the publisher's web‐site:

**Fig. S1.** Physiological data for the transition experiment from 150% to 40% aerobiosis. At time zero, the input gas composition was changed from 7.5% v/v oxygen (150% aerobiosis) to 1.88% oxygen (40% aerobiosis). A. Change in optical density at 600 nm. B. Corresponding increase in the specific acetate excretion rate. C. Dry weight at the high and low aerobiosis conditions and at 300 min after the transition. D. Cell viability measured by plate counts. SS; steady‐state. The data represent the means of at least three determinations; error bars are standard deviation from the mean.
**Fig. S2.** Summary of gene expression changes during the 150–40% aerobiosis transition. Genes were divided into up‐ or downregulated based on a twofold or more change in expression and their functions classified using the TIGR main roles categories. The full data set from which this figure is derived is given in Supporting Information Table S1.
**Fig. S3.** Measurements of substrate dependent respiratory activities. Cells from the steady‐states indicated were harvested from the chemostat, washed and resuspended in 20 mM phosphate buffer pH 7.4 and incubated with the substrates indicated in A–E (10 mM final concentration for all except sulphite which was used at 0.5 mM final concentration) at 37°C in a Clark type oxygen electrode. For formate oxidation (A), cells were grown to steady‐state either in media without added selenate (standard medium used for all other experiments in this study) or with 10 μM sodium selenate as indicated. In (F), the activity of the cytochrome *c* oxidase CcoNOQP was assayed as oxygen consumption in the presence of 1 mM sodium ascorbate and 0.25 mM *N,N,N′,N′*‐ tetramethyl‐*p*‐phenylenediamine (TMPD). The histograms represent the means of three determinations; error bars are standard deviation from the mean. **** *p* < 0.0001; *, *p* < 0.05 by Students *t*‐test. ns, not significant.
**Fig. S4.** Comparison of *c*‐type cytochrome abundance at 40% and 150% aerobiosis. Cell‐free extracts (∼200 μg protein) were denatured in Laemmli sample buffer without mercaptoethanol, run on SDS‐PAGE gels which were either stained with Coomassie Blue (A) or blotted onto nitrocellulose where *c*‐type cytochromes were detected by their haem‐associated peroxidase activity using enhanced chemiluminescence with CCD imaging (B). The designations of those *c*‐type cytochromes shown are based on their molecular weights and comparison with the pattern of cytochromes in the gels published previously (Liu and Kelly, 2015). Lanes 1–3 for each aerobiosis condition are independent replicate steady‐state samples.
**Fig. S5.** Modelling of RacR activity. A. Inferred activity of RacR during the 150–40% aerobiosis shift. A connectivity matrix for RacR (see Experimental Procedures) was used as the input for TFINFER, which interrogated the microarray dataset for the transition. RacR activity is predicted to show a transient decrease up to 60 min after the transition. B. Histogram of the relative regulatory strengths of target genes in the RacR regulon.
**Fig. S6.** Pattern of glycosylated proteins at 40% and 150% aerobiosis. Glycosylated proteins were detected after SDS‐PAGE and electroblotting onto nitrocellulose membranes by reaction with Soybean agglutinin lectin conjugated with horseradish peroxidase (SBA‐HRP), followed by enhanced chemiluminescence and CCD imaging. A. Coomassie blue stained gel with ∼200 μg protein loaded per lane. B. Corresponding blot after reaction with SBA‐HRP. Lanes 1–3 for each aerobiosis condition are independent replicate steady‐state samples.

**Table S1.** Microarray data and RT‐PCR comparing the 150% and 40% aerobiosis steady states and at time points during the transition from 150% to 40% aerobiosis. In the ‘All significant (*p* < 0.05) genes’ tab, the fold‐changes of genes which showed a greater than twofold change in expression with a significance of *p* < 0.05 at any one of the time points or in a steady‐state are listed in a simple coloured heat map format (shades of red indicate > 2.0‐fold up‐regulation and shades of blue indicate > 2.0‐fold down‐regulation). Fold‐changes less than twofold are shown as greyed out values. A few values were not significant at *p* < 0.05 and are highlighted in red. The ‘summary’ tab just shows the significant twofold changes in either direction. The ‘RT‐PCR’ tab, shows the results of RT‐PCR on the same steady‐state samples as used for the microarray, for the *mfrA, peb1a, cstA, putA* and *aspA* genes, using the primers shown.

**Table S2.** Label‐free LC‐MS/MS analysis of proteins at 40% and 150% aerobiosis. Samples from three independent steady‐states at 1.88% v/v oxygen or 40% aerobiosis (LF1‐3) and three at 7.5% v/v oxygen or 150% aerobiosis (LF‐4‐6) were analysed by LC‐MS/MS as described in Experimental Procedures. In the tables shown, A,B,C are gel lane replicates of each independent steady‐state sample. The emPAI values shown in these columns are the average for triplicated Orbitrap injections for each gel lane replicate. The ‘normalised emPAI HB sorted’ tab sorts the data according to significance based on the Hochberg‐Benjamini correction, with *p* values <0.02. The ‘normalised emPAI Cj sorted’ tab sorts the data based on Cj locus tag/gene number. The ‘normalised emPAI ratio sorted’ tab sorts the data on the basis of the ratio of the means of the LF1A‐LF3C data and the LF4A‐LF6C data to give a fold‐change of protein abundance between 40% and 150% aerobiosis. In each case, the identified proteins are grouped according to whether they are highest at 40% or 150% aerobiosis. Data for proteins detected by LC‐MS/MS but showing no statistically significant change and proteins not detected but predicted from the genome sequence, are also shown. The ‘Venn Diagram’ tab shows a simple Venn diagram to illustrate the numbers of differentially expressed proteins under each condition.

**Table S3.** 2D‐gel analysis of proteins from cells grown at all steady‐states from 25% to 345% aerobiosis. In the ‘All steady‐state raw data’ tab, the normalised spot volumes from image analysis for a given protein are shown for multiple gels run with a minimum of 1 steady‐state per oxygen input condition (50% and 75% aerobiosis) or with two or more independent steady‐states (25%, 40%, 88%, 100%, 150%, 200% and 345% aerobiosis). CV refers to coefficient of variation. The data are sorted by the ANOVA value. Note that some proteins appeared as multiple spots and so may have more than one entry. For each aerobiosis condition the spot volumes for a given protein on each gel were averaged and these values are shown in the ‘mean spot volumes’ tab. These mean values were used to construct the graphs of changes in selected protein abundance shown in the paper.
