# Supplementary material for: Transcriptome and proteome dynamics in chemostat culture reveal how Campylobacter jejuni modulates metabolism, stress responses and virulence factors upon changes in oxygen availability
Source: Environ Microbiol. 2017 Oct 2;19(10):4326–48. doi: 10.1111/1462-2920.13930 (PMC5656828; doi:10.1111/1462-2920.13930)
Supplement: Supplementary file 1 — Fig. S1. Physiological data for the transition experiment from 150% to 40% aerobiosis. At time zero, the input gas composition was changed from 7.5% v/v oxygen (150% aerobiosis) to 1.88% oxygen (40% aerobiosis). A. Change in optical density at 600 nm. B. Corresponding increase in the specific acetate excretion rate. C. Dry weight at the high and low aerobiosis conditions and at 300 min after the transition. D. Cell viability measured by plate counts. SS; steady‐state. The data represent the means of at least three determinations; error bars are standard deviation from the mean. Fig. S2. Summary of gene expression changes during the 150–40% aerobiosis transition. Genes were divided into up‐ or downregulated based on a twofold or more change in expression and their functions classified using the TIGR main roles categories. The full data set from which this figure is derived is given in Supporting Information Table S1. Fig. S3. Measurements of substrate dependent respiratory activities. Cells from the steady‐states indicated were harvested from the chemostat, washed and resuspended in 20 mM phosphate buffer pH 7.4 and incubated with the substrates indicated in A–E (10 mM final concentration for all except sulphite which was used at 0.5 mM final concentration) at 37°C in a Clark type oxygen electrode. For formate oxidation (A), cells were grown to steady‐state either in media without added selenate (standard medium used for all other experiments in this study) or with 10 μM sodium selenate as indicated. In (F), the activity of the cytochrome c oxidase CcoNOQP was assayed as oxygen consumption in the presence of 1 mM sodium ascorbate and 0.25 mM N,N,N′,N′‐ tetramethyl‐p‐phenylenediamine (TMPD). The histograms represent the means of three determinations; error bars are standard deviation from the mean. **** p < 0.0001; *, p < 0.05 by Students t‐test. ns, not significant. Fig. S4. Comparison of c‐type cytochrome abundance at 40% and 150% aerobiosis. Cell‐free extracts (∼20 [file EMI-19-4326-s001.pdf]

Transcriptome and proteome dynamics in chemostat culture reveal how *Campylobacter jejuni* modulates metabolism, stress responses and virulence factors upon changes in oxygen availability

Edward Guccione[1], John J. Kendall[1], Andrew Hitchcock[1], Michael White[1], Nitanshu Garg[1], Francis Mulholland[2] , Robert K. Poole[1], and David J. Kelly[1*]

Supplementary Figures S1- S6.

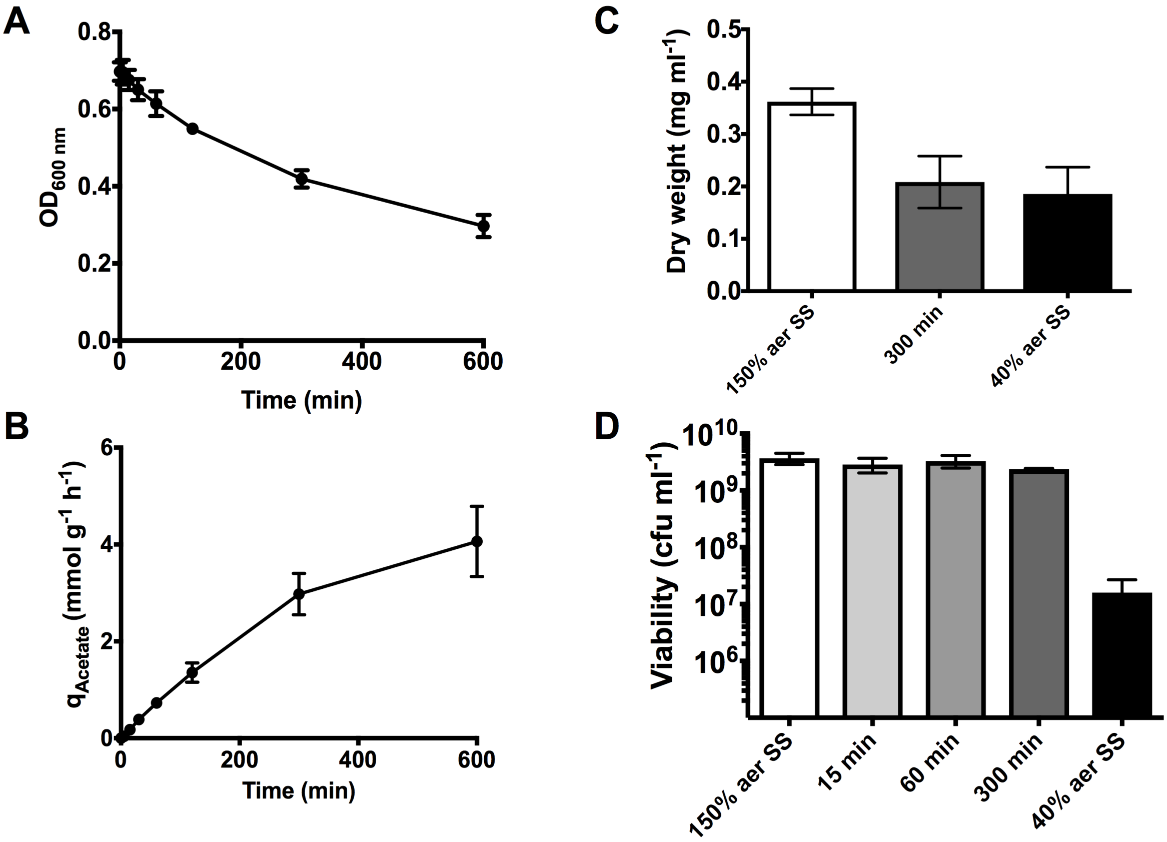

Figure S1. **Physiological data for the transition experiment from 150% to 40% aerobiosis**. At time zero, the input gas composition was changed from 7.5% v/v oxygen (150% aerobiosis) to 1.88% oxygen (40% aerobiosis). **(A)** Change in optical density at 600 nm. **(B)** Corresponding increase in the specific acetate excretion rate. **(C)** Dry weight at the high and low aerobiosis conditions and at 300 min after the transition. **(D)** Cell viability measured by plate counts. SS; steady-state. The data represent the means of at least three determinations; error bars are standard deviation from the mean.

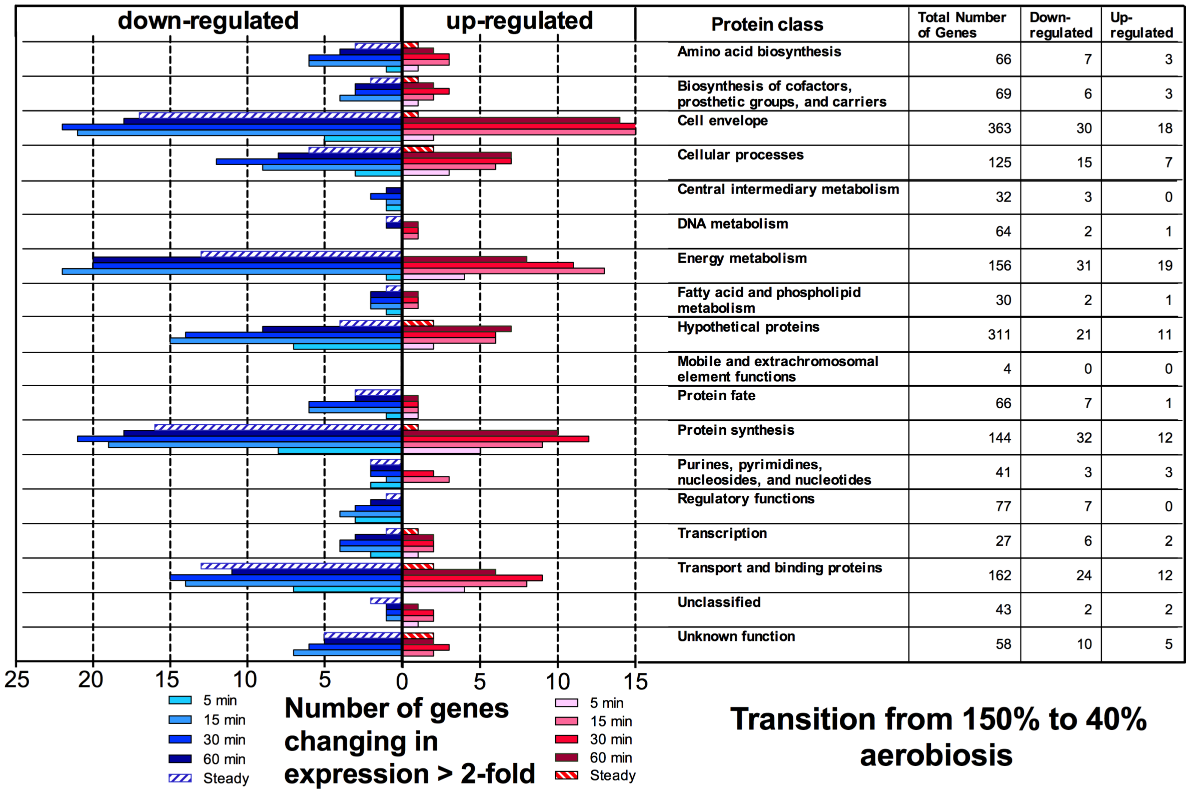

Protein class	Total Number of Genes	Down-regulated	Up-regulated
Amino acid biosynthesis	66	7	3
Biosynthesis of cofactors, prosthetic groups, and carriers	69	6	3
Cell envelope	363	30	18
Cellular processes	125	15	7
Central intermediary metabolism	32	3	0
DNA metabolism	64	2	1
Energy metabolism	156	31	19
Fatty acid and phospholipid metabolism	30	2	1
Hypothetical proteins	311	21	11
Mobile and extrachromosomal element functions	4	0	0
Protein fate	66	7	1
Protein synthesis	144	32	12
Purines, pyrimidines, nucleosides, and nucleotides	41	3	3
Regulatory functions	77	7	0
Transcription	27	6	2
Transport and binding proteins	162	24	12
Unclassified	43	2	2
Unknown function	58	10	5

Transition from 150% to 40% aerobiosis

Figure S2. Summary of gene expression changes during the 150% to 40% aerobiosis transition. Genes were divided into up- or downregulated based on a 2-fold or more change in expression and their functions classified using the TIGR main roles categories. The full data set from which this figure is derived is given in Table S1.

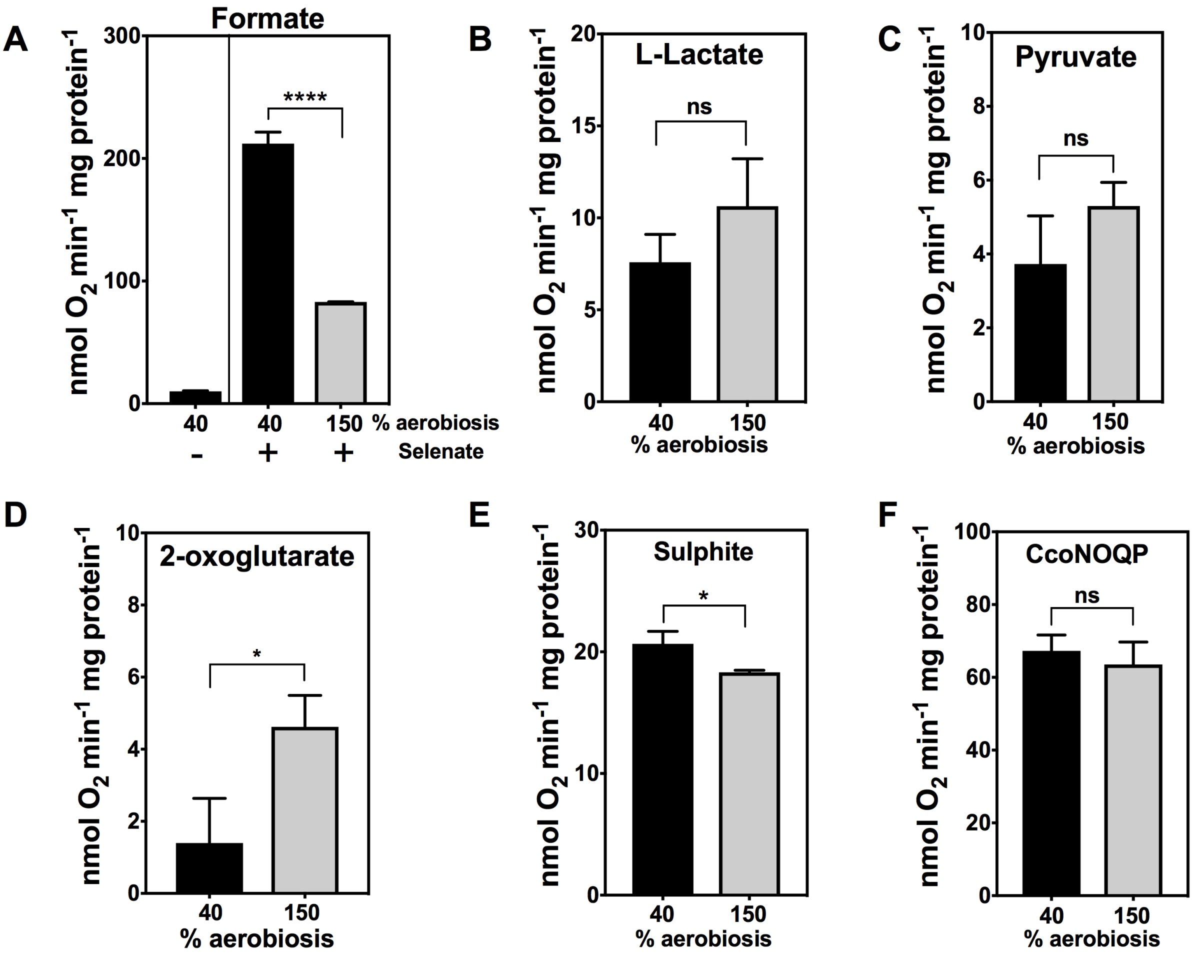

Figure S3. Measurements of substrate dependent respiratory activities. Cells from the steady-states indicated were harvested from the chemostat, washed and resuspended in 20 mM phosphate buffer pH 7.4 and incubated with the substrates indicated in A-E (10 mM final concentration for all except sulphite which was used at 0.5 mM final concentration) at 37 °C in a Clark type oxygen electrode. For formate oxidation (A), cells were grown to steady-state either in media without added selenate (standard medium used for all other experiments in this study) or with 10 μM sodium selenate as indicated. In (F), the activity of the cytochrome *c* oxidase CcoNOQP was assayed as oxygen consumption in the presence of 1 mM sodium ascorbate and 0.25 mM *N,N,N',N'*- tetramethyl-*p*-phenylenediamine (TMPD). The histograms represent the means of three determinations; error bars are standard deviation from the mean. **** $p < 0.0001$; *, $p < 0.05$ by Students t-test. ns, not significant.

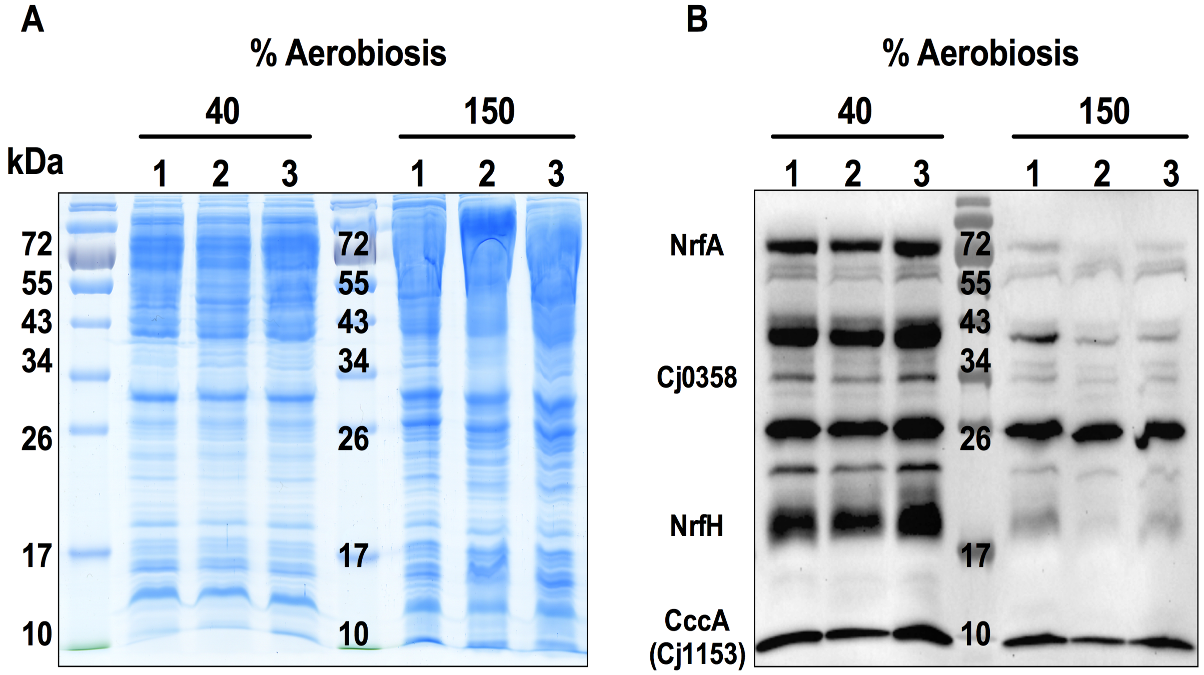

Figure S4. **Comparison of *c*-type cytochrome abundance at 40% and 150% aerobiosis**. Cell-free extracts (~200 µg protein) were denatured in Laemmli sample buffer without mercaptoethanol, run on SDS-PAGE gels which were either stained with Coomassie Blue **(A)** or blotted onto nitrocellulose where *c*-type cytochromes were detected by their haem-associated peroxidase activity using enhanced chemiluminescence with CCD imaging **(B)**. The designations of those *c*-type cytochromes shown are based on their molecular weights and comparison with the pattern of cytochromes in the gels published previously (Liu and Kelly, 2015). Lanes 1-3 for each aerobiosis condition are independent replicate steady-state samples.

.

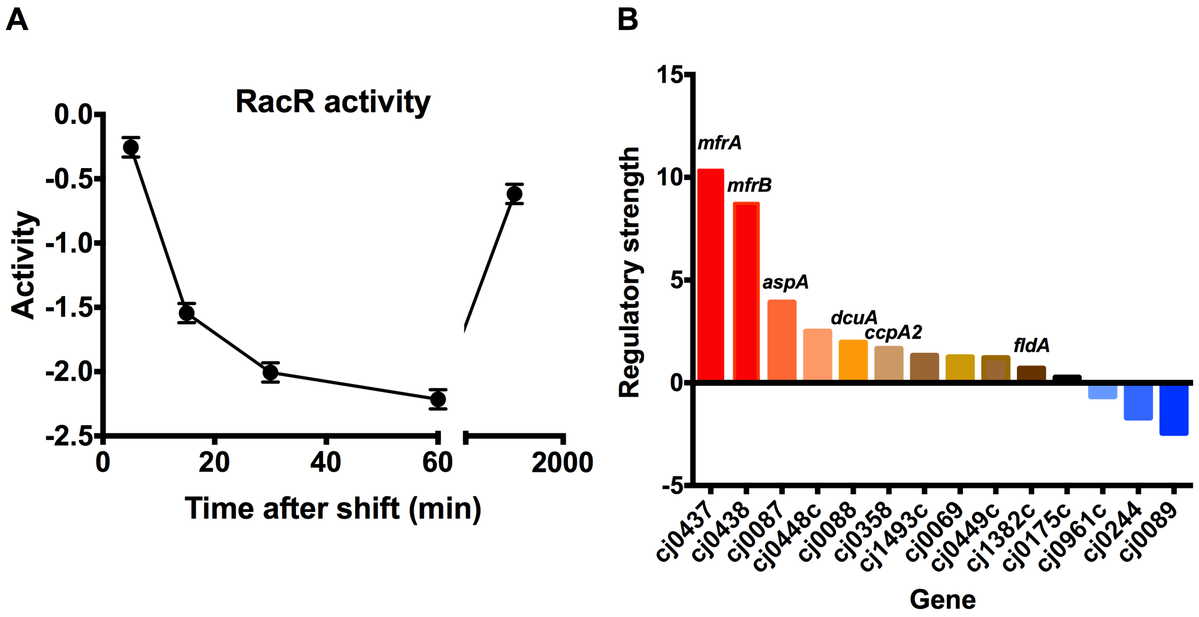

Figure S5. Modelling of RacR activity. **(A)** Inferred activity of RacR during the 150% to 40% aerobiosis shift. A connectivity matrix for RacR (see Experimental Procedures) was used as the input for TFINFER, which interrogated the microarray dataset for the transition. RacR activity is predicted to show a transient decrease up to 60 min after the transition. **(B)** Histogram of the relative regulatory strengths of target genes in the RacR regulon.

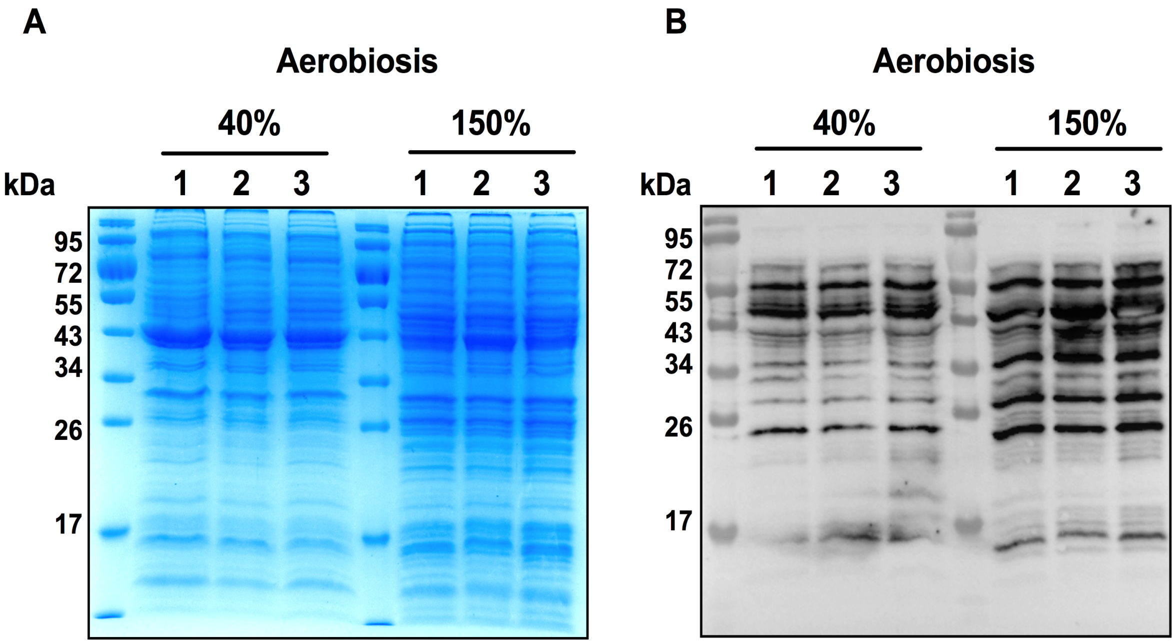

Figure S6. **Pattern of glycosylated proteins at 40% and 150% aerobiosis.** Glycosylated proteins were detected after SDS-PAGE and electroblotting onto nitrocellulose membranes by reaction with Soybean agglutinin lectin conjugated with horseradish peroxidase (SBA-HRP), followed by enhanced chemiluminescence and CCD imaging. **(A)** Coomassie blue stained gel with ~200 µg protein loaded per lane. **(B)** Corresponding blot after reaction with SBA-HRP. Lanes 1-3 for each aerobiosis condition are independent replicate steady-state samples.
